# The role of E3 ubiquitin ligases in bone homeostasis and related diseases

**DOI:** 10.1016/j.apsb.2023.06.016

**Published:** 2023-07-06

**Authors:** Yuechao Dong, Yangshan Chen, Guixing Ma, Huiling Cao

**Affiliations:** Department of Biochemistry, School of Medicine, Southern University of Science and Technology, Guangdong Provincial Key Laboratory of Cell Microenvironment and Disease Research, Key University Laboratory of Metabolism and Health of Guangdong, Shenzhen 518055, China

**Keywords:** E3 ubiquitin ligase, UPS, Osteogenesis, Bone resorption, Osteoblast, Osteoclast, Chondrocyte, PROTAC

## Abstract

The ubiquitin–proteasome system (UPS) dedicates to degrade intracellular proteins to modulate demic homeostasis and functions of organisms. These enzymatic cascades mark and modifies target proteins diversly through covalently binding ubiquitin molecules. In the UPS, E3 ubiquitin ligases are the crucial constituents by the advantage of recognizing and presenting proteins to proteasomes for proteolysis. As the major regulators of protein homeostasis, E3 ligases are indispensable to proper cell manners in diverse systems, and they are well described in physiological bone growth and bone metabolism. Pathologically, classic bone-related diseases such as metabolic bone diseases, arthritis, bone neoplasms and bone metastasis of the tumor, etc., were also depicted in a UPS-dependent manner. Therefore, skeletal system is versatilely regulated by UPS and it is worthy to summarize the underlying mechanism. Furthermore, based on the current status of treatment, normal or pathological osteogenesis and tumorigenesis elaborated in this review highlight the clinical significance of UPS research. As a strategy possibly remedies the limitations of UPS treatment, emerging PROTAC was described comprehensively to illustrate its potential in clinical application. Altogether, the purpose of this review aims to provide more evidence for exploiting novel therapeutic strategies based on UPS for bone associated diseases.

## Graphical abstract

E3 ubiquitin ligases regulate bone homeostasis and pathological events variously. The UPS-dependent therapeutic significance of bone-associated diseases has been synthetically expatiated.

**Figure:**
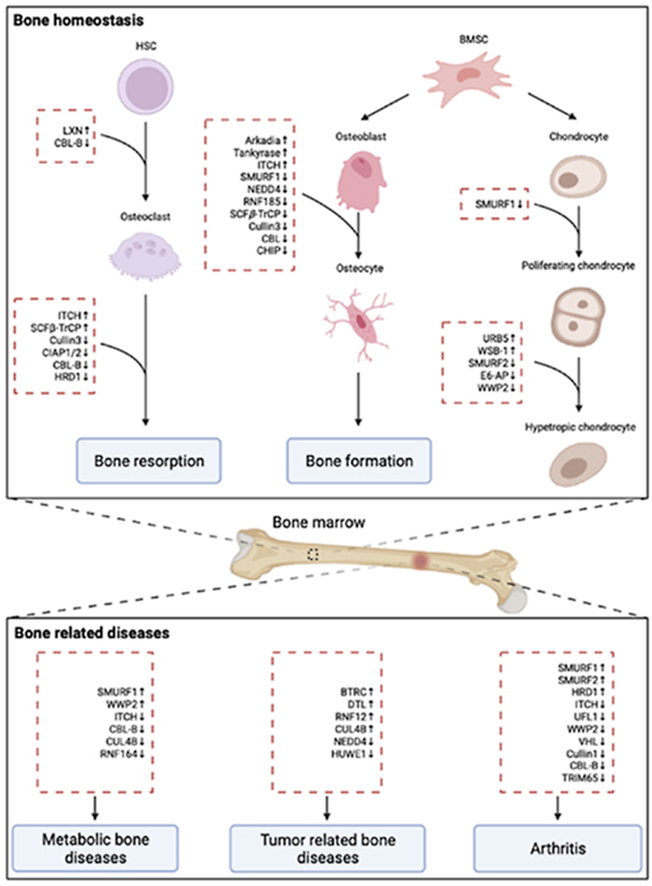

## 1 Introduction

The muti-functional cells or even the healthy whole organism is the consequence of proteostasis, which requires the intricate interactions between protein synthesis and degradation. Continuity of both processes derives from alteration effect of the proteasomes *via* replacing pre-existing proteins with new ones in order to ensure proper states of cells^1^. Generally, mistakes are considered to occur for once among 20,000 amino acids during protein synthesis^2^. Moreover, protein degradation is also indispensable for the regulation of cell proliferation, differentiation and apoptosis, which manifests as the recycling of amino acids and removing of improperly folded proteins to avoid the error responses^3^.

**Figure 1:**
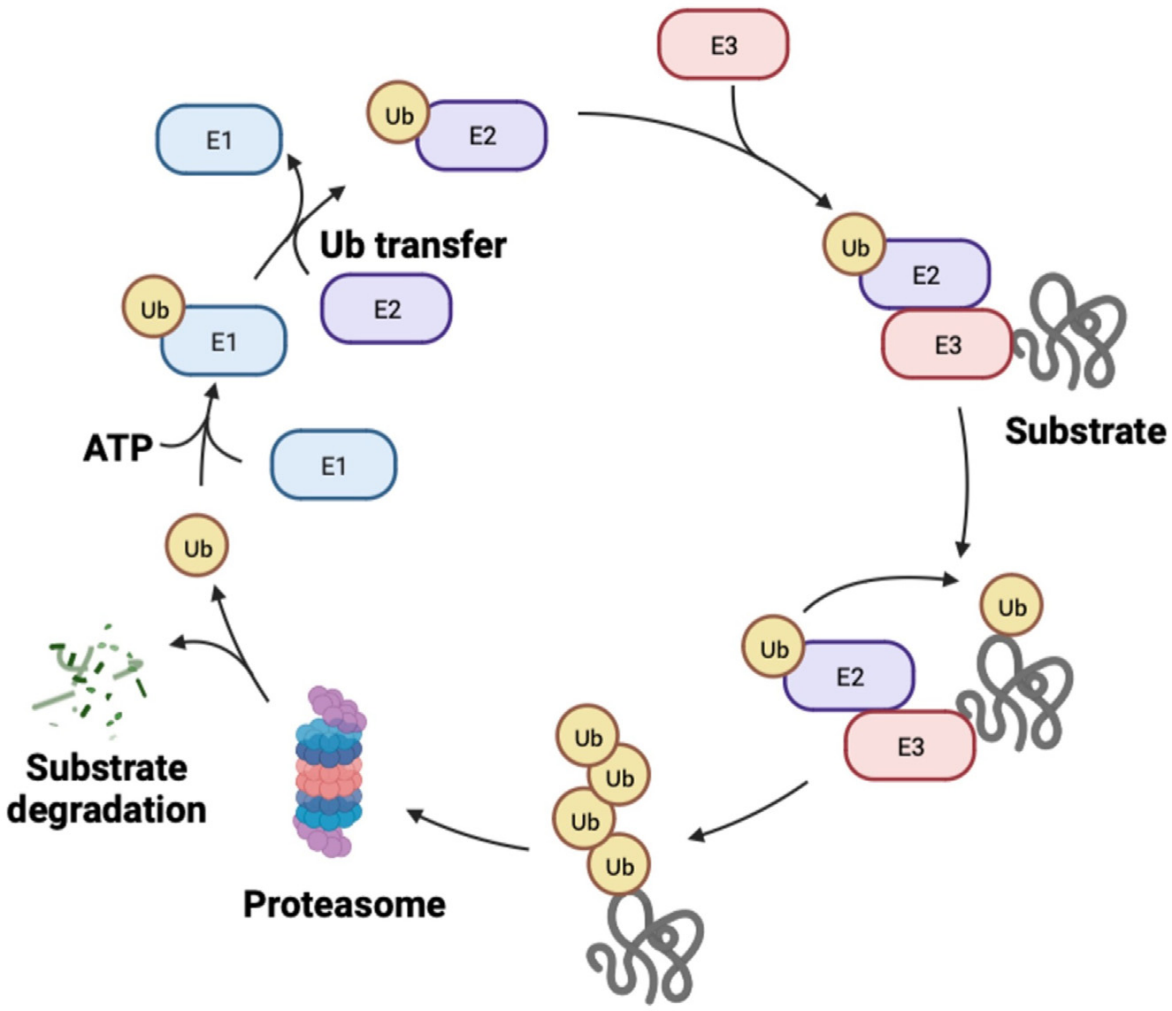
Protein degradation process mediated by UPS. The covalent interaction between one ubiquitin molecule and the ubiquitin-activating enzyme E1 is carried out by means of the energy from adenosine triphosphate (ATP). Then ubiquitins (Ubs) can be transported to ubiquitin-conjugating enzyme E2 and accompanied by the departure of E1. E2 can form a complex with E3 ubiquitin ligase and substrates. Being equipped with the complex, Ubs can be transported to E3 and then onto the substrate. Successive Ub molecules are provided to form poly-Ub chains and consequently, proteins tagged by Ubs are recognized and degraded by proteasomes. Dissociative Ubs are recycled for a new round reaction of ubiquitination.

Ubiquitin–proteasome system (UPS) is responsible for the degradation of over 80% proteins selectively *in vivo*^4^. Ubiquitins (Ubs) are small molecular proteins with 76 amino acids which exist in eukaryotes extensively. They can be connected by enzymatic reactions to mark and degrade target proteins in a covalent attachment mode termed as ubiquitination^5^. In the case of energy supplied from adenosine triphosphate (ATP), alternative polyadenylation of one Ub molecule is induced by ubiquitin activating enzyme E1 and then the affected Ub molecule is transferred to the cysteine residue of the E1 active center. Based on this, E1 is qualified for transferring Ubs to the cysteine residue of ubiquitin-conjugating enzyme E2. Finally, highly conserved E3 ubiquitin ligases combine to the substrates directly and specially to catalyze the Ub molecules, which are transferred from E2 to target proteins. The polyubiquitinated proteins above are recognized by proteasomes to facilitate unfolding and deubiquitinating. Finally, Ub molecules are intactly liberated by de-ubiquitinating enzymes for the following cycle of reaction^6^ (Fig. 1). Of course, target proteins suffered from ubiquitination modification are not all degraded and participate other biological progresses such as DNA repair, endocytosis, autophagy, transcription, immunity and inflammation^7^.

**Figure 2:**
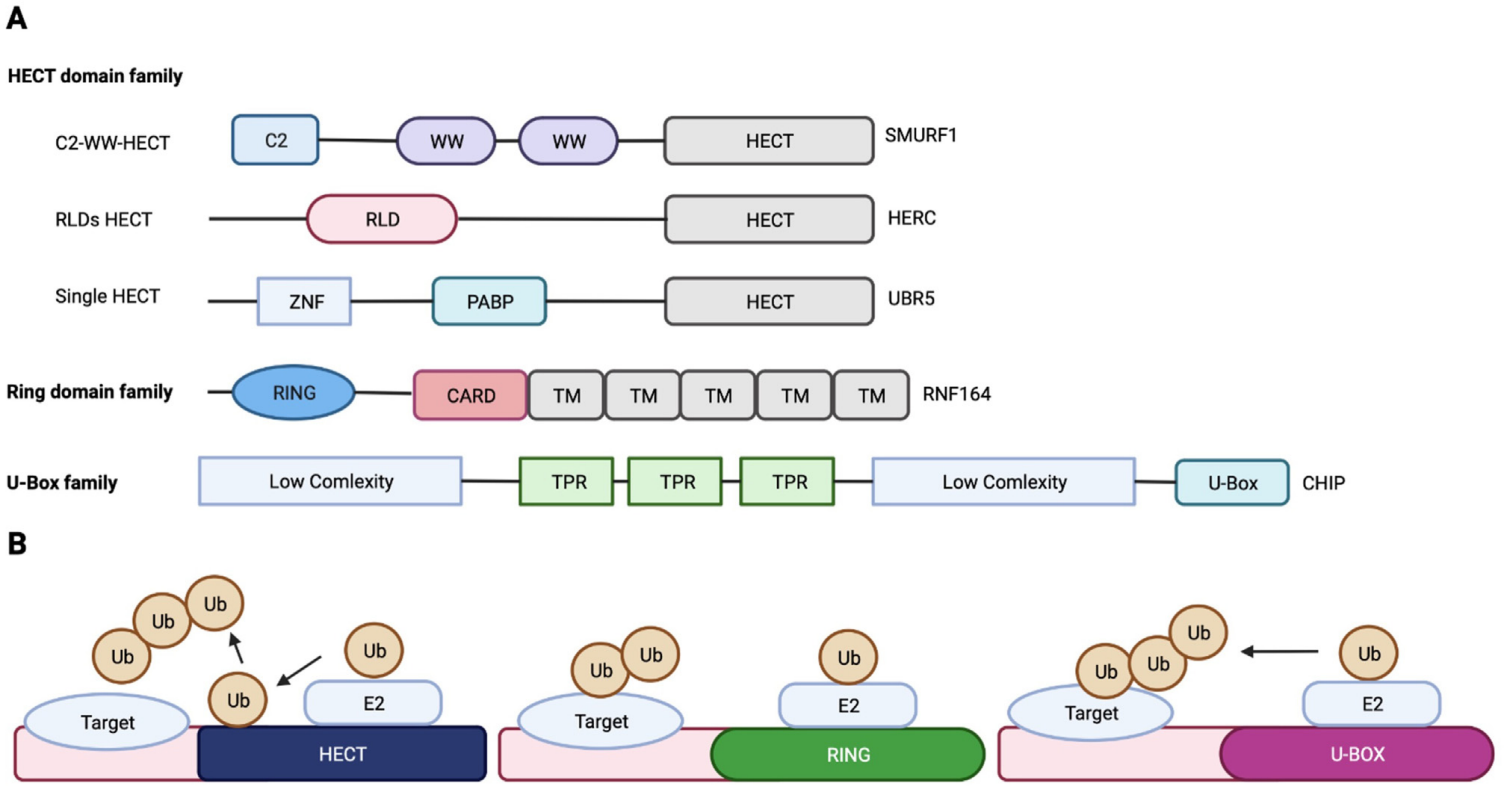
Domain architecture of E3 ligases of different families and their effect on ubiquitins. (A) According to different domain architectures, E3 ligases are classified into homologous to E6-associated protein at the carboxy-terminus (HECT) type E3 ligases, RING type E3 ligases and U-box type E3 ligases. HECT type E3 ligases are further classified into 3 subunits and one representative member of all the families or subunits is provided to exhibit the domain character. (B) Ubs are linked to HECT domains *via* ubiquitin-conjugating enzyme E2 and then transferred to substrates. However, the ring or U-box structure domain submits Ubs to substrates directly without attaching them.

E3 ligases are usually classified into 3 big families: homologous to E6-associated protein at the carboxy-terminus (HECT) domain family, RING domain family and U-box family. For HECT family members, there are cysteines in conserved C-terminals which interact with Ubs by thioester bonds to transfer E3 to substrates. Although the involvement of conserved C-terminals in catalytic action is valid, only N-terminal domains determine specification of the target proteins and classify these ligases into three further subfamilies: C2-WW-HECT with WW domains and Tryprophan–Tryprophan domains; HERC with RCC1-like domains; single-HECT without any domains above. The two lobes of one E3 ligase affiliated to the HECT family are bound by flexible hinges to modulate the direction of Ub transfer^8^^,^^9^. However, RING domain families contain cysteines and histidines combine with two Zn^2+^ ions to form spherical structures. These E3 ligases can transfer Ubs from ubiquitin-conjugating enzyme E2 to substrates directly but without combing Ubs^10^. Monomers, homodimers or heterodimers of RING domains can come into play. Similarly, U-box domains are folded equally with RING domains and also present as monomers or homodimers. But there is no Zn^2+^ ion in this structure. The domain architecture of E3 ligases and their effect on Ub molecules are revealed in Fig. 2. So far, a large number of scholars focus their research on various E3 ligases, and UPS dependency of cell behaviors such as proliferation, differentiation and apoptosis have already been well established^11^. The multi-function derives from the modification effect from different Ubs. In terms of mechanisms, seven K residues and *α*-amino group on the N-terminal methionine 1 of Ubs are able to be ubiquitinated by other Ub molecules to generate polyUb chains^12^.

As one of the largest organs which occupies around 10%–14% of the total body weight, bones mainly support the posture and movement of the bodies. Skeleton system approximately consists of 90% bone matrix and 10% functional cells such as osteoblasts (OBs), osteoclasts (OCs), osteocytes and chondrocytes^13^. Bone metabolism is highly dynamic and bone tissue is continuously renewed during the whole lifetime. Therefore, all bones undergo complete renovation around each decade^14^. The homeostasis above has always subordinated to external forces or internal signal pathways, and accumulating researches expound the implication of E3 ligases in bone remodeling through recognizing different target proteins^15^. Based on that, we summarized the roles of E3 ligases on the fine-tuning of bone homeostasis, such as bone formation, bone resorption and chondrogenesis in this review. Moreover, the clinical relevance of E3 ligases regulation on orthopedic disorders was also expatiated to provide an insight into developing therapeutic strategies.

## 2 The role of E3 ubiquitin ligases in bone formation

**Figure 3:**
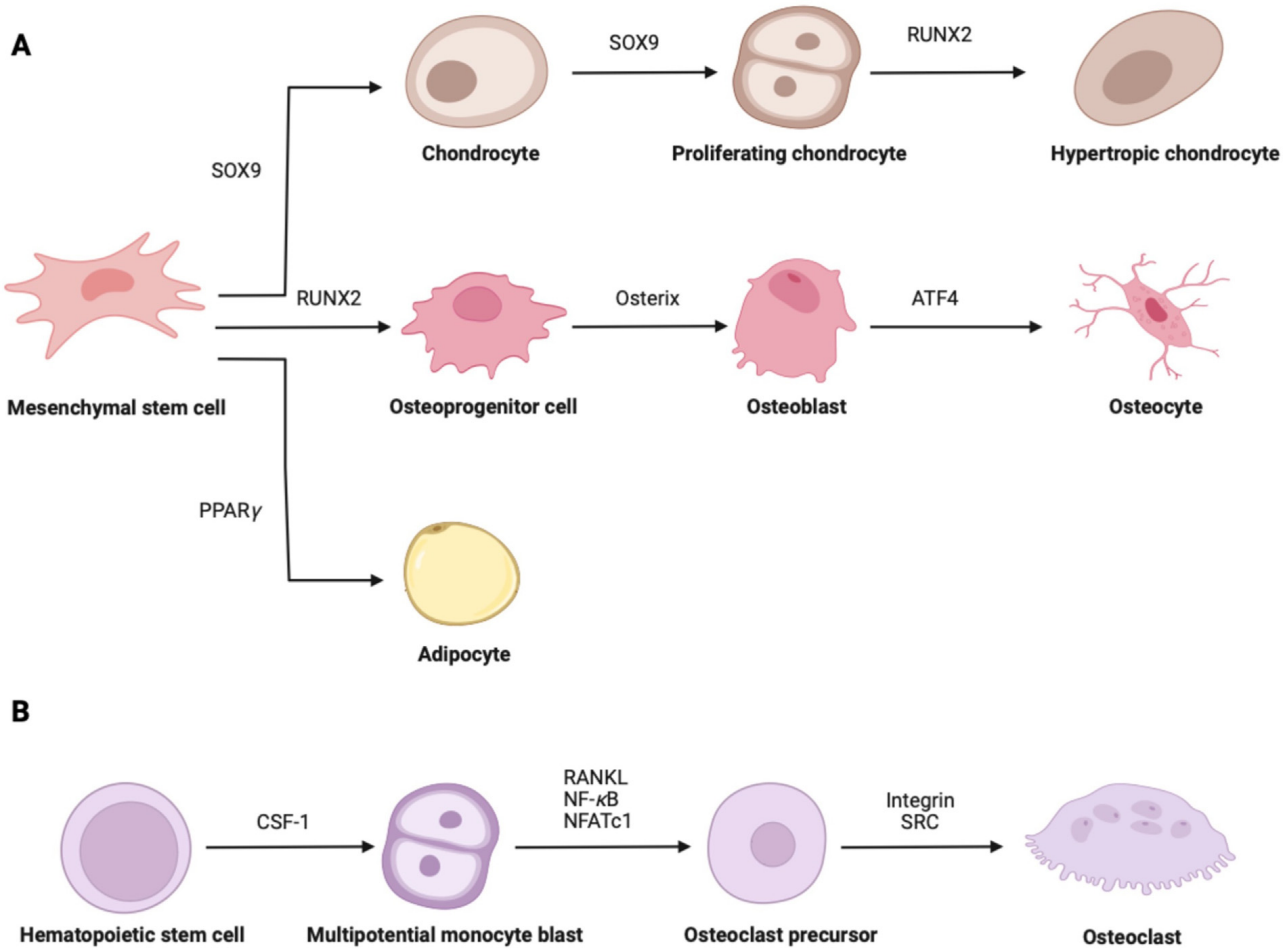
Functional cells involved in bone homeostasis originate from the multipotent stem cells in a well-ordered manner. Bone homeostasis and development require functional harmony among osteoblasts, chondrocytes, adipocytes and osteoclasts. (A) Multipotent mesenchymal stem cells **(**MSCs**)** rich in SRY-box transcription factor 9 (SOX9) can differentiate into proliferative chondrocytes, which followed by Runt-related transcription factor 2 (RUNX2) -induced cellular hypertrophy. However, RUNX2 promotes MSCs to turn into osteoprogenitor cells, the stem cells located in skeletal system and can differentiate into osteoblasts for bone formation. As the terminal product of osteoblastic lineage, the generation of osteocytes is beneficial from Activating transcription factor 4 (ATF4) positive OBs. And pathologically, overexpression of peroxisome proliferator activated receptor *γ* (PPAR*γ*) initializes the appearance of adipocytes. (B) Osteoclast formation depends on the proliferative and differential hematopoietic stem cells. Finally, under the functions of Integrin or SRC, the intermediate products can generate mature OCs.

Mesenchymal stem cells (MSCs) are multi-potential stem cells which can differentiate into adipocytes, chondrocytes and OBs, etc. under different conditions (Fig. 3A). Runt-related transcription factor 2 (RUNX2) facilitates MSCs differentiate into osteoprogenitor cells, which further differentiate into cells express OBs phenotypic genes by virtue of Osterix. Terminally, OBs differentiate into osteocytes which are embedded in bone matrix to integrate physical stimuli and biological reaction^16^. The osteogenic differentiation of MSCs is orchestrated by multiple signal pathways, cytokines or growth factors such as growth hormone (GH)^17^, parathyroid hormone (PTH)^18^, insulin-like growth factor (IGF)^19^, fibroblast growth factor (FGF)^20^, bone morphogenetic protein (BMP)^21^, Wnt/*β*-Catenin^22^, and Notch pathway^23^, etc.

Over the recent years, the osteogenic regulation competence of UPS has been gradually confirmed and arouses more attention. As the major functional cells responsible for osteogenesis, differentiation of OBs lineage at different stages are mediated by the interaction between E3 ligases and their target proteins.

### 2.1 E3 ubiquitin ligases regulating BMP signaling pathway

Being located on cellular surface, both type I and type II BMP receptors are organized into homomeric dimers. Type II receptors bind to BMP ligands primarily and form tetrameric complexes with phosphorylated type I receptors. Osteogenic competence of BMP2 and BMP7 has been vastly studied on OBs lineage^24^. In the process above, phosphorylation of the downstream Smad1/5/8 proteins contributes to the combination with Smad4 to initiate the expression of RUNX2 and Osterix. As the markers of osteogenesis, expression of target genes downstream of these transcription factors such as Alkaline Phosphatase, Collagen type 1, Osteocalcin and Osteopontin, etc., promotes OBs differentiation^25^^,^^26^. As transforming growth factor-*β* (TGF-*β*) family members, BMPs also regulate non-Smad pathways such as mitogen-activated protein kinases (MAPKs) *via* protein kinase D to mediate OBs differentiation^27^^,^^28^. Interestingly, under the initiation of exogenous BMPs, activated Smads and MAPK pathways converge to facilitate the expression of RUNX2 and direct the OBs lineage differentiation^29^.

Smad ubiquitination regulatory factor 1 (SMURF1), is primarily verified to mediate the degradation of Smads and inhibit OBs differentiation^30^. Moreover, the trans-membrane BMP receptors are also known to suffer from cysteine-residue substitution and lysosomal degradation when being exposed to active SMURF1 and the ligase-inactive SMURF1 displays opposite effect to maintain the BMP activity on the cell membrane. As the best studied member of C2-WW-HECT E3 ligase, SMURF1 was revealed to capture Smad1/5 by WW domains. The interaction above is mediated by the proline–tyrosine motif of Smads and terminates the BMPs-induced OBs differentiation^31^.

The roles of SMURF1 on non-Smad pathways are also demonstrated in preosteoblasts or mature OBs. This phenomenon is ascribed for the degradation of MAPKs and JUNB mediated by SMURF1^32^^,^^33^. Contradictorily, although both have the phosphorylation effect on MAPKs, there is antagonistic effect between the epidermal growth factor (EGF) and BMPs, which is manifested as the proliferation ability conferred by EGF restricts the BMPs-induced OBs differentiation. In EGF pathway, combination of transcription factors such as c-Jun and RUNX2 to SMURF1 promoter in turn degrades RUNX2 and forms negative feedback^34^. As previously described, BMP-induced osteogenesis profits from Smad6 deficiency, which is possible to block the negative feedback between SMURF1 and RUNX2. In conclusion, as the most extensively explored E3 ligase compared with other members during the BMP-induced osteogenic processes, the predominating effect of SMURF1 has been illustrated in almost each phase.

Although recombinant human BMP is clinically applied on spinal fusion surgery and fracture repair, the individual discrepancy of local bone formation and the underlying mechanisms are not well understood^35^. Among aging osteoporotic mice classified into normal BMP9/elevated SMURF1 and decreased BMP9/normal SMURF1, the former present lower level of RUNX2, p-Smad1, serum Osteocalcin and relatively more evident bone loss. As the SMURF1 inhibitor, chalcone derivative 2-(4-cinnamoylphenoxy) acetic acid targets conjugate oligopeptide (AspSerSer) 6 to make the systemic osteogenesis in aging osteoporotic mice^36^. Therefore, SMURF1 is more influential to osteogenesis due to their effects on some other pathways, which will be illustrated further in the following context.

Besides SMURF1, at the early stage of osteogenesis, WW domain containing E3 ubiquitin protein ligase 1 (WWP1) and Itchy E3 ubiquitin protein ligase (ITCH) also modulate BMP pathway by degrading JUNB and conversely, WWP1-null or ITCH-null mice exhibit increasing serum Osteocalcin level, bone formation and mineral apposition rate^37^^,^^38^. Under the inflammatory stimulation such as tumor necrosis factor-*α* (TNF-*α*), increased Hsc70-interacting protein (CHIP) was induced to recognize K55 and K386 residues of Osterix as the ubiquitinating sites. Therefore, CHIP limitation in OBs may be a new mechanism to prevent osteoporosis during inflammatory arthritis^39^. Casitas B-lineage lymphoma proto-oncogene-B (CBL-B) and Casitas B lineage lymphoma (C-CBL) are also verified to negatively regulate the BMP-induced osteogenesis by reducing the stability and transcription activity of Osterix^40^.

On the contrary, some other E3 ligases enable the OBs differentiation. Transgenic mice with Collagen1*α*1 (Col1*α*1) promoter-driven neuronal precursor cell-expressed developmentally downregulated 4 (NEDD4) expression in immature OBs exhibit promoted osteogenesis, and mice with conditional knockout of NEDD4 show low bone mass. The enhancing OBs proliferation during prosperous osteogenesis is caused by NEDD4-induced p-Smad1 abrogation and p-Smad2 potentiation at the early stage^41^. And Arkadia, a RING-type E3 ligase, amplifies the BMP pathway through interacting with multiple domains of the BMP-specific suppressors such as Smad6^42^.

### 2.2 E3 ubiquitin ligases regulating Wnt signaling pathway

WNT ligands combine the co-receptor of Frizzled and lipoprotein receptor-related proteins 5/6 (LRP5/6), then recruit Dishevelled (DVL) to transfer the phosphorylation effect of Casein kinase 1 on LRP5/6. Moreover, Glycogen synthase kinase-3*β* is dissociated from AXIN which allows the entry of *β*-Catenin into nucleus. Then *β*-Catenin together with T-cell factor/lymphoid enhancer-binding factor (TCF/LEF) facilitates target genes expression which are crucial to osteogenesis^22^. Under fundamental states, intracellular free *β*-Catenin is recognized and folded by Axin and adenomatous polyposis coli, followed by the exposure of several phosphorylation sites^43^. Then Casein kinase 1 is recruited to phosphorylate Ser45 of *β*-Catenin and three other sites Thr41, Ser37 and Ser33 are also phosphorylated by the Glycogen synthase kinase-3*β*^44^. Phosphorylated *β*-Catenin is subjected to E3 ligase mediated ubiquitination and degraded by 26 S proteasomes.

Skp1-Cul1-F-box protein complex containing the F-box protein *β*-TRCP (SCF*β*-TrCP) with the ring structure is the first identified E3 ligase with *β*-Catenin degradation effect in Hela cells^45^. During OBs differentiation, BMP-2 promotes *Lrp5* gene expression and suppresses SCF*β*-TrCP to alleviate *β*-Catenin degradation. The synergistic effects between BMP and WNT/*β*-Catenin pathways lie on reduced SCF*β*-TrCP^46^^,^^47^. Consequently, WNT/*β*-Catenin and BMP-2 pathways can be regulated collectively by the same E3 ligases. EGF/SMURF1 pathway is identified to inhibit osteogenesis by degrading *β*-Catenin^48^. Moreover, the E3 ubiquitin ligase ring finger protein 185 (RNF185) in MSCs leads to the degradation of DVL2 and the OBs differentiation is suppressed. The terminated differentiation of OBs can be resumed by the re-activated DVL2^49^. On the contrary, WNT/*β*-Catenin is enhanced by RNF146, the Ring E3 ligase which directly interacts with tankyrase through its WWE domain and attaches adenosine-5′-diphosphate-ribose polymers onto AXIN, the *β*-Catenin destruction complex^50^. Mice deficient of RNF146 suffer from impaired bone development and osteopenia caused by glucose intolerance^51^.

### 2.3 E3 ubiquitin ligases regulating other osteoblastogenesis pathways

FGF receptors (FGFR) are transmembrane and tyrosine kinase receptors (RTK) which combine with the ligands to induce the cross-phosphorylation of tyrosine residues in the intracellular domain to direct OBs differentiation^52^. The phosphotyrosine binding domain of E3 ligase C-CBL interacts with activated RTK and restrains the osteogenic differentiation of MSCs. The interaction is disrupted by CBL inhibitor which enhances FGFR2 and OBs markers. The osteogenic differentiation of MSCs can be strengthened *via* activation of MAPKs or phosphatidylinositol-3-kinase/protein kinase B (PI3K/AKT) pathway^53^.

Indian Hedgehog (IHH) activates GLI family transcription factors which display both transcriptional activation and suppression effects on target genes to promote endochondral bone development^54^. As the surface receptor recognized and activated by ligands, patched removes the inhibitory effect on Smoothened (SMO) to activate GLI2 activator (GLI2A) and prevent formation of GLI3 repressor (GLI3R). Cullin-ring E3 ligase recognizes substrates GLI2, full-length GLI3 and GLI3R by the Speckle-type POZ subunit (SPOP). Loss of SPOP in limb mesenchyme results in enhanced GLI3 but not GLI2 to cause shorter limbs in mice, which is rescued by GLI3 deprivation. Therefore, SPOP may prefer GLI3 to GLI2^55^. The normalization of OBs differentiation needs the coexistence of SPOP and IHH pathway.

In the early stage of osteogenesis, OBs differentiation is promoted by the close collaboration and mutual promotion between BMP pathway and Hedgehog^29^. And SCF*β*-TrCP in OBs precursor ubiquitinates p-GLI2 and downregulates BMP-2 to hamper OBs differentiation^56^.

In contrast to pathways promoting bone formation, highly conserved Notch pathway has the opposite effect. As the receptor combines with corresponding ligands, Notch is dissociated to release the Notch intracellular domain, which can be translocated into nucleus and regulate transcription of target genes, such as *Hey1* and *Hes1,* to inhibit OBs differentiation^57^. ITCH, an E3 ligase of HECT family, binds to the N-terminal of Notch intracellular domain through WW domains and promotes Notch ubiquitination *via* HECT domain. ITCH deficiency leads to reduced MSCs differentiation into OBs and results in osteopenia phenotype of mice^58^^,^^59^.

PTH is synthesized and secreted by parathyroid and functions on skeleton, renal and intestinal systems. Alteration of PTH continuity selectively activates distinct E3 ligases and determines the osteogenic manner of OBs. Under the treatment of continuous PTH, RUNX2 deficiency induced by enhanced SMURF1 contributes to the loss of anti-apoptosis ability and the impairment of bone formation^60^. Conversely, activating transcription factor 4 can be induced by intermittent PTH to remove the proteasomal degradation of another E3 ligase SCF*β*-TrCP on osteogenic pathways^38^, thus enhancing bone formation.

### 2.4 E3 ubiquitin ligases regulating RUNX2, the common substrate of osteogenic pathways

RUNX2 is also gradually demonstrated as the direct substrate of SMURF1. However, Proline–Tyrosine motif of RUNX2 is not necessary to the SMURF1-mediated RUNX2 degradation and the process above can be enhanced by the interaction between Smad6 and RUNX2^61^. Moreover, as the prerequisite of functioning on RUNX2, SMURF1 should be phosphorylated by adenosine 5′-monophosphate-activated protein kinase on serine 148 in advance^62^. The mRNA level of *Smurf1* is also modulated by various transcription factors such as CREB regulated transcription coactivator 2, Activator protein 1 and RUNX2, which are stimulated by TNF-*α*^63^^,^^64^. The involvement of SMURF1 in RUNX2 and Osterix degradation is also deciphered under the stimulation of TNF-*α* although the direct interaction between SMURF1 and Osterix still remains unclear^65^. On the contrary, SMURF1 deficiency only increases RUNX2 at protein level but not mRNA level and manifests deubiquitination on the target protein. Hence, there is a regulatory circuit between RUNX2 and SMURF1, which is verified not only in OBs, but also in human dental pulp stem cells^66^.

**Figure 4:**
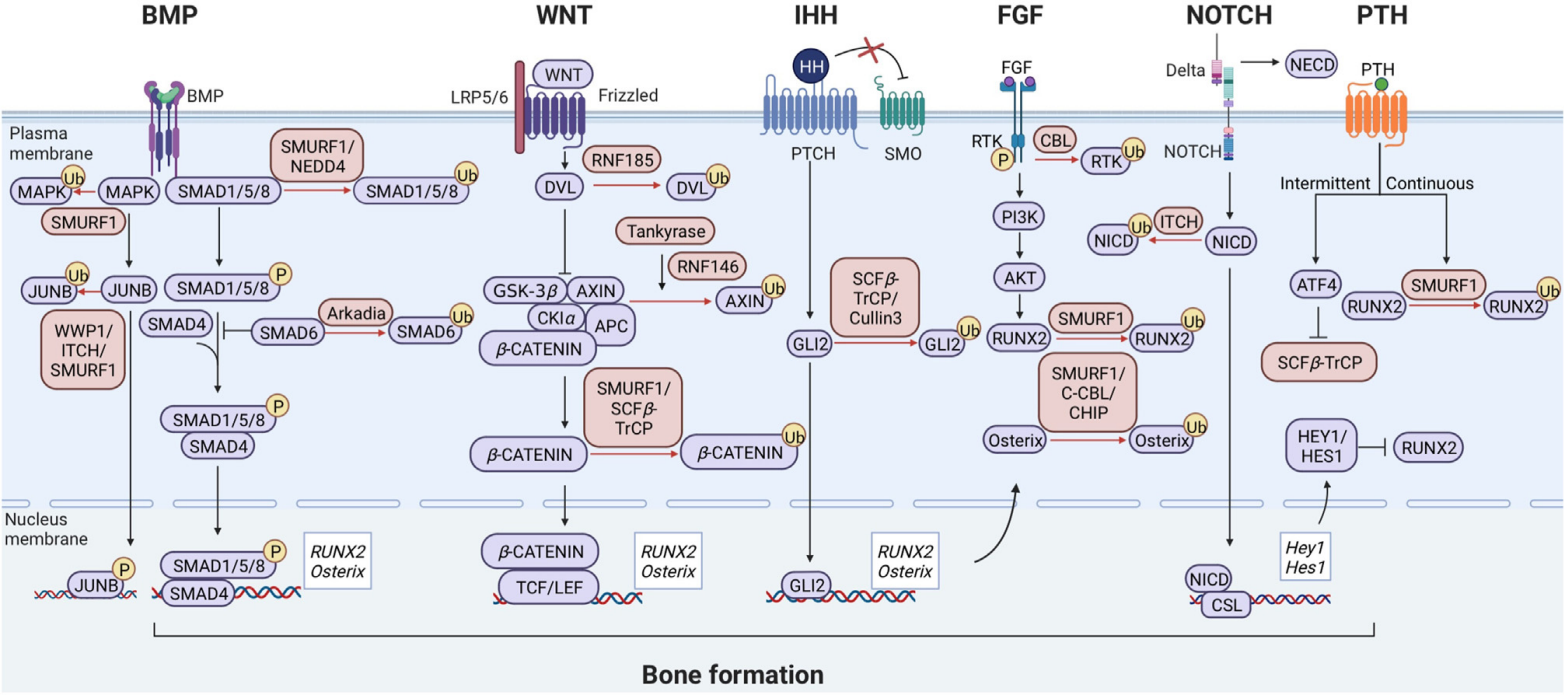
The participation of E3 ubiquitin ligases in osteogenic pathways. A complicated network constructed by E3 ligase and signal pathways is indispensable for proper osteogenesis and is well described below. (A) Bone morphogenetic protein (BMP) pathway: Smad ubiquitination regulatory factor 1 (SMURF1) negatively regulates BMP pathway *via* functioning on BMP receptors and its effect protein SMAD1/5. Moreover, Smad families also provide targets for other E3 ligase such as neuronal precursor cell-expressed developmentally downregulated 4 (NEDD4) and Arkadia to control BMP pathway. Conversely, BMP also inhibits Skp1–Cul1–F-box protein complex containing the F-box protein *β*-TRCP (SCF*β*-TrCP) and rescues the impairment of osteogenesis through other reactions. SMURF1 inhibits non-SMAD pathway by targeting Mitogen-activated protein kinases (MAPKs) and JunB. WW domain containing E3 ubiquitin protein ligase 1 (WWP1) and Itchy E3 ubiquitin protein ligase (ITCH) inhibit non-SMAD pathway by targeting JUNB. (B) Wnt/*β*-Catenin pathway: RNF185 prevents Wnt pathway *via* degrading Dishevelled 2 (DVL2). Both Smurf1 and SCF*β*-TrCP interact and ubiquitinate *β*-Catenin to play the inhibitory roles. However, RNF146 mediates the PARsylation of Tankyrase to stimulate Wnt pathway *via* degrading Axin. Finally, *β*-Catenin is dissociated. (C) Indian Hedgehog (IHH) pathway: GLI proteins in Hedgehog pathway are objects of SCF*β*-TrCP and Cullin3. (D) Fibroblast growth factor (FGF) pathway: Casitas B-lineage lymphoma (CBL) blocks FGF pathway at the receptor level. In a word, As the terminal products of the pathways above, Runt-related transcription factor 2 (RUNX2) can be directly ubiquitinated by SMURF1 and Osterix is also degraded by SMURF1, CBL and Hsc70-interacting protein (CHIP). (E) Notch pathway: ITCH terminates Notch pathway *via* binding the N-terminal portions of Notch intracellular domain (NICD) segments. (F) Parathyroid hormone (PTH) pathway: continuous PTH inhibits SCF*β*-TrCP and intermittent PTH promotes Smurf1. OBs differentiation is decided by the PTH continuity.

In brief, the effect of E3 ligase is well established in various osteogenic pathways associated with OBs behaviors (Fig. 4). Compared with the activator of single osteogenic pathway, targeting specific E3 ligase manifests more promising efficiency *via* emancipating more approaches for osteogenesis promotion. The research focuses on E3 ligase is potential to improve the strategic precision and efficiency of therapies towards abnormal OBs differentiation and osteogenesis during metabolic bone diseases.

## 3 The role of E3 ubiquitin ligases in bone resorption

The continuous bone regeneration occurred during remodeling can be initiated by bone resorption, during which the bone matrix is decomposed and absorbed by the acids and enzymes produced by OCs^67^, which originate from hemopoietic stem cells (HSCs) and locate on the bone surface (Fig. 3B)^68^. OCs secrete proteases, tartrate resistant acid phosphatase and acid ions to degrade the inorganic mineral substance and absorb the degradation products by pinocytotic vesicles for further degradation.

Colony-stimulating factor-1, also known as macrophage colony-stimulating factor (M-CSF), promotes MAPKs or PI3K/AKT pathway to mediate the differentiation of HSCs into OCs precursor^69^^,^^70^. Then mature OCs are generated under the induction of the receptor activator of nuclear factor kappa-B (NF-*κ*B) ligand (RANKL)^71^. The activation effect of RANKL on NF-*κ*B is mediated by TNF receptor-associated factor 6 (TRAF6), the ubiquitination effect of which takes down the inhibitor of NF-*κ*B (I*κ*B*α*) from NF-*κ*B and allows the entrance of NF-*κ*B into nucleus to activate c-Fos expression, c-Fos then activates the promotors of nuclear factor of activated T cells 1 (NFATC1), the downstream target of c-Fos^72^. Moreover, the enhancing effect of TRAF6 on c-Jun and c-Fos is also mediated by MAPK cascades. In stark contrast to the direct ubiquitination effect of E3 ligases, TRAF6 and transforming growth factor-*β*-activated kinase 1 form complex to mediate the downstream MAPK/NF-*κ*B, which increases osteoclastogenesis^73^. There are also negative regulators such as osteoprotegerin, which combines RANKL and inhibits the differentiation of OCs^74^. Given to the fact that bone resorption is determined by complicated pathways, it supplies numerous potential target proteins for UPS which has been thoroughly explored in OCs.

### 3.1 E3 ubiquitin ligases regulating canonical pathways of osteoclastogenesis

**Figure 5:**
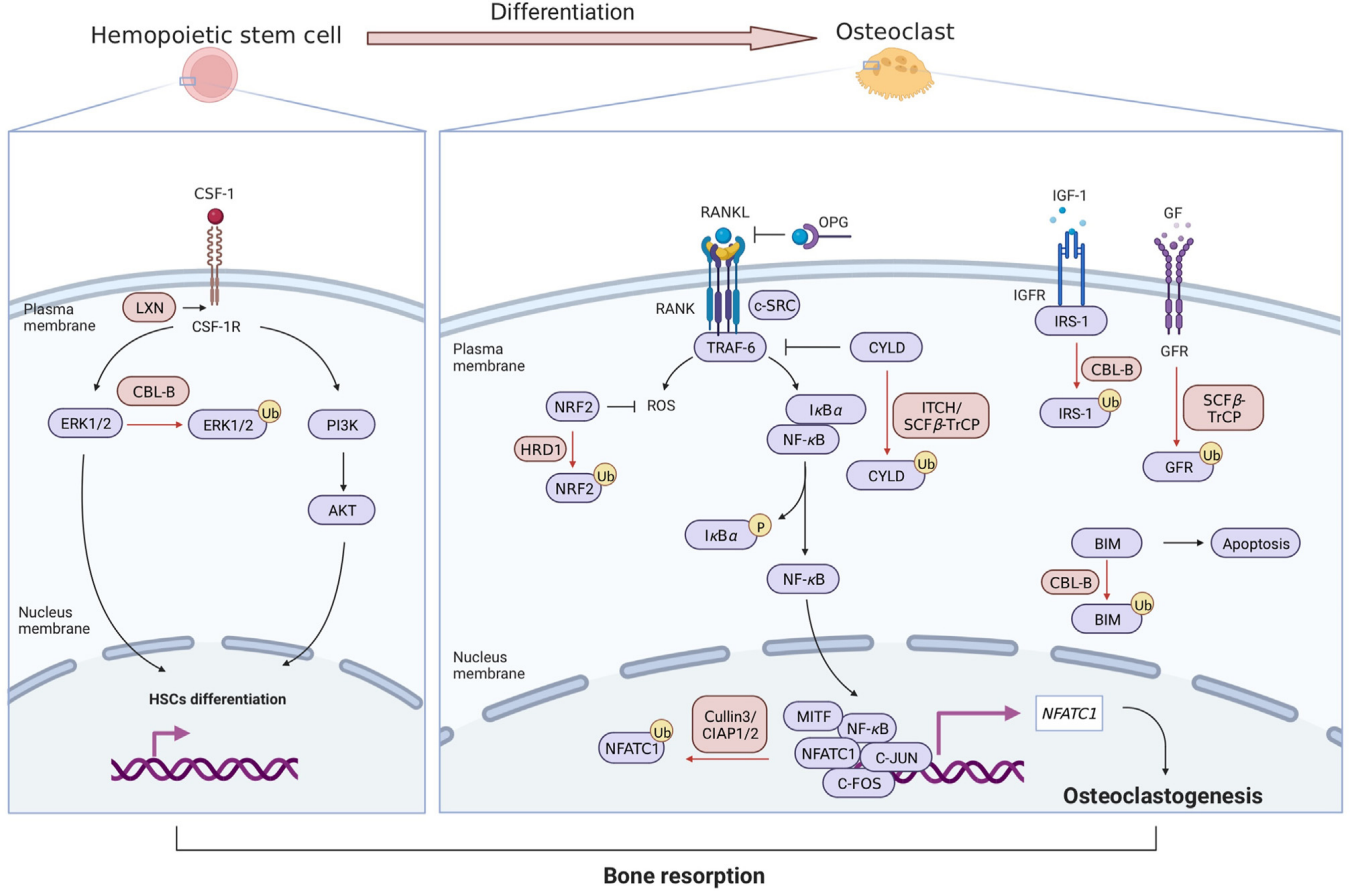
Osteoclast differentiation, survival and hypertrophy are also regulated by E3 ubiquitin ligases. (A) In hematopoietic stem cells (HSCs), extracellular-signal-regulated kinase 1/2 or phosphatidylinositol-3-kinase/protein kinase B (PI3K/AKT) phosphorylated by colony-stimulating factor-1 (CSF1) facilitates the differentiation to osteoclasts, during which can be accelerated by the E3 ligase ligand of numb protein-X 2 (LNX2) and CBL-B. (B) Receptor activator of nuclear factor kappa-B ligand (RANKL) pathway: the upstream protein TRAF-6 can be inhibited by Cylindromatosis (CYLD). E3 ligase ITCH and SCF*β*-TrCP act on CYLD to perform disinhibition on the pathway. Similarly, E3 ligase HMG-CoA reductase degradation protein 1 (HRD1) ubiquitinates nuclear factor erythroid 2-related factor 2 (NRF2) to abolish the anti-ROS action and RANKL pathway also remains unimpeded. The terminal product nuclear factor of activated T cells 1 (NFATC1) is responsible for OCs maturation and also degraded by E3 ligases Cullin3 and The cellular inhibitor of apoptosis proteins 1 and 2 (cIAP1/2). (C) OCs activity also depends on the recognition between hormones and relevant receptors. E3 ligase CBL-B ubiquitinates insulin receptor substrate (IRS-1) and SCF*β*-TrCP ubiquitinates growth hormone (GH) receptors. (D) CBL-B ubiquitinates BIM to prevent the apoptosis of OCs.

E3 ligases participate in the osteoclastogenesis from HSCs to mature OCs (Fig. 5). Ligand of numb protein-X 2 is described as the ring E3 ligase containing PDZ domain (full name is post-synaptic density-95, disks-large and zonula occludens-1 domain) and regulates RANKL/NF-*κ*B and M-CSF/Extracellular-signal-regulated kinase/AKT pathways to prompt the osteoclastogenesis in the early stage. However, the underlying mechanism remains to be identified^75^. E3 ligases C-CBL and CBL-B in OCs regulate cell migration and bone resorption^15^. Overexpression of CBL-B decreases OCs activity by regulating both NF-*κ*B and Extracellular-signal-regulated kinase signaling pathways. The osteopenia phenotype of CBL-B^−/−^ mice is caused by increased bone resorption and unaltered osteogenesis^76^. However, C-CBL promotes OCs survival by ubiquitynating BIM, a pro-apoptotic BH3-only Bcl-2 family member^77^. Interestingly, BIM mutation mice suffered from slight osteopetrosis although owing increased OCs number^78^. The mechanism of function shortage remains unclear so far and may probably be due to the impairment of podosome-like structures which create the acidic environment for bone resorption.

In response to RANKL/RANK, Lys63-linked polyubiquitination on TRAF6 in OCs can be inhibited by cylindromatosis (CYLD). The phosphorylation of CYLD at Ser432/Ser436 enables the binding of SCF*β*-TrCP and then TRAF6 is ubiquitinated to retain the cellular functions^79^. Although SCF*β*-TrCP is extensively studied as the I*κ*B*α* inhibitor in various cell types, the relationship between SCF*β*-TrCP and I*κ*B*α* has not been studied in OCs^45^^,^^80^^,^^81^, and the dual mechanisms of SCF*β*-TrCP on NF-*κ*B indicate its potential effects in OCs. TRAF6 functions as a ubiquitin ligase and the Lys63-linked polyubiquitination of TRAF6 facilitates bone resorption. ITCH/CYLD interaction prevents the ubiquitination of TRAF6 to impose restrictions on OCs. ITCH mutation mice exhibit increased OCs number and bone resorption ability caused by inhibiting TRAF6 deubiquitination^82^. On the contrary, transcription factor NF-*κ*B is also the inhibitory upstream of RNF146 and regulates the stability of SRC and 3BP2, the adaptor protein essential for OCs^83^.

Cellular inhibitors of apoptosis proteins 1 and 2 is the first E3 ligase reported to ubiquitinates NFATC1 in the late stage of osteoclastogenesis^84^. As another RING-finger type E3 ligase, Cullin3 recognizes NFATC1 *via* combining the protein tyrosine phosphatase domain of Kelch repeat and BTB domain-containing protein 11, the specific adaptor of Cullin3 and deficiency of which also favors OCs functions^85^. One recent work reported that OCs maturation is benefited from inhibition of interaction between CBL and NFATC1^86^.

### 3.2 E3 ubiquitin ligases regulating Nrf2 pathway

Nuclear factor erythroid 2-related factor 2 (NRF2) implements cellular protection in multi aspects and therefore has attracted extensive study^87^. OCs activity can be amplified by the stimulation of oxidative stress to promote bone resorption. As the classical antioxidant enzyme, NRF2 in OCs prevents the OCs differentiation by regulating RANKL and NF-*κ*B^88^. HMG-CoA reductase degradation protein (HRD1) is the sole determinant E3 ligase which mediates the degradation of NRF2 in OCs to guarantee bone resorption, which can be inhibited by Hrd1 inhibitor Octylitaconate^89^. However, in other cell types, the KEAP1/CUL3/RBX1 complex and SCF*β*-TrCP can also present NRF2 to proteasomes, the inhibition of which manifests as the intensive NRF2/antioxidant responsive element (ARE) pathway^90^. However, the effect of HRD1 in NRF 2 degradation during osteoclastogenesis need further studies.

### 3.3 The effect of E3 ubiquitin ligases on hormone pathways

GH and IGF-1 facilitate bone resorption by stimulating the pre-existing OCs. In contrast to some OCs like cell types, the blockade of IGF-1 abolishes the effect of GH on OCs entirely^91^. CBL-B mediates Insulin receptor substrate degradation and confers the IGF-1 resistance to OCs. And CBL-B knockdown enhances IGF-I signal^92^. WD40 domains of SCF*β*-TrCP mediate the endocytosis and degradation of GH receptors through binding to ubiquitin-dependent endocytosis motif in the cytokine receptor box 2 region^93^. Above all, E3 ligases also regulate the response of OCs to hormones in various manners.

### 3.4 E3 ubiquitin ligases regulating podosome belts assembled by integrin/SRC pathway

As the hub between cytoskeleton and extracellular matrix (ECM), *α*v*β*3 integrin locates on the membrane of OCs and ensures the cellular attachment to bone matrix. Moreover, cytoskeletal organization of OCs modulated by integrins is necessary to cell migration and the generation of sealing zone^94^. The adaptor protein SRC is *α*v*β*3 integrin-dependent and promotes bone resorption competence mainly *via* modulating podosome assembly^95^. Podosomes are actin rich structures located on the ventral membrane of diverse cells including OCs. Functional units of podosomes are bipartite structures consisting of a core structure, full of actin filaments and actin associated proteins, and a ring structure which contains integrins and focal adhesion proteins. In OCs, podosome units are organized into belt like superstructure to form the sealing zone, which surrounds and labels the bone resorption area^96^. The acidic environment convenient for the degradation of bone matrix is beneficial to the matrix metalloproteases (MMPs) recruited by podosomes. It is demonstrated that podosome assembly on osteoclasts depends highly on SRC-CBL pathway in a positive feedback way. SRC phosphorylates C-CBL at tyrosine residues, which in turn activates SRC through binding to the SRC homology 2 (SH2) domains. Consequently, both SRC and C-CBL are reinforced by the reciprocal action above. Osteopetrosis accompanied with weakened podosomes in OCs results from deubiquitination of SRC or C-CBL^97^.

In summary, the signal pathways associated with bone resorption functions are also regulated comprehensively by different E3 ligases. Physiologically, the fine tuning of bone turnover is inseparable from abundant UPS systems. Moreover, the disturbance of bone homeostasis largely results from aberrant alteration of any UPS system and highlights the clinical value of each E3 ligase in developing therapeutic strategies.

### 3.5 E3 ubiquitin ligases regulating pathways underlying osteoblasts-osteoclasts communication

Bone metabolism depends closely on the intercommunication between OBs and OCs, which regulates the behaviors of each other by direct contact or protein secretion. The crucial roles of E3 ligases in osteoblast-osteoclast communication are illustrated as follows.

OCs degrade bone matrix and release latent growth factors to regulate OBs. The osteogenic effect of IGF is blocked by CBL-B-induced ubiquitination of insulin receptor substrate-1^92^. The anti-osteogenic effect of TGF-*β* is mediated by enhanced SMURF1 and ubiquitination of RUNX2^32^. OBs also produce M-CSF and RANKL to initiate bone resorption in different phases of OCs. In response to both Smad and non-Smad pathways of BMP stimulation, RANKL expression is increased in OBs to initiate bone resorption by recognizing RANK in OCs^98^. As previously mentioned, Nedd4 ubiquitinates P-Smad1^41^, SMURF1 ubiquitinates P-Smad 1/5/8 and MAPKs^31^^,^^32^, Arkadia ubiquitinates Smad6 to relieve P-Smad 1/5/8^42^ and WWP1/ITCH ubiquitinates JUNB^37^^,^^38^, all are important for RANKL expression. Therefore, plenty of E3 ligases may regulate the expression of RANKL in OBs to mediate OCs behaviors.

EphrinB2 is the ligand on OCs surface and recognized by EPHB4, the receptor on OBs, to promote OBs differentiation through MAKPs pathway^99^. In turn, EphrinB2/EPHB4 blocks the osteoclastogenic c-Fos/NFATC1 cascade. EphrinB2 is the target gene of NFATC1^100^. As mentioned above, Cellular inhibitors of apoptosis proteins 1/2 and Cullin3 ubiquitinate NFATC1 which may prevent the mRNA expression of EphrinB2^84^^,^^85^. Therefore, the interaction from OCs to OBs can be cut off by the E3 ligases. Semaphorin 3 A (SEMA3A) is the ligand on OBs surface and binds to neuropilin-1 (NRP1), the receptor on OCs, to inhibit RANKL-induced OCs differentiation. In turn, SEMA3A/NRP1 promotes OBs differentiation through the WNT/*β*-Catenin pathway^101^. In OBs, FGF/FGFR phosphorylates RTK to active PI3K/AKT, thus increases downstream SEMA3A expression. The E3 ligase CBL ubiquitinates RTK and may inhibit SEMA3A expression, thus abolishes the promoting effect of OBs on OCs^53^^,^^102^. Collectively, E3 ligases most likely participate in the crosstalk between OBs and OCs. E3 ligases with dual effects on bone formation and resorption are more worthy of clinical study.

## 4 The role of E3 ubiquitin ligases play on chondrogenesis

Adult cartilage mainly consists of chondrocytes and collagen tissues but not vessels and nerves^103^. These tough connective tissues enhance the flexibility of articulations and play protective and supportive roles during movement. Chondrogenesis is the multi-step process which provides structural foundation for the following osteogenesis^104^. MSCs can differentiate into osteoprogenitor cells and then chondrocytes. Chondrocytes are embedded in the cartilage matrix, the components of which such as COL2a1 and Aggrecan can be secreted by chondrocytes themselves. Then cartilage rudiments are forged to generate long bone-like structures to facilitate the following osteogenesis^105^. Finally, under the manipulation of RUNX2, chondrocytes turn into hypertrophic cells which produce COLX, MMPs, aggrecanases and vascular endothelial growth factor A (VEGF) and promote degradation of cartilage matrix^106^. Osteoprogenitor cells in the deep layer differentiate into osteoblasts and produce osteoid to calcify the bone collar on the surface and mid piece of cartilage rudiment^107^.

Chondral differentiation and development are dominated by classical pathways such as TGF-*β*/Smad, BMP, WNT/*β*-Catenin, Notch and IHH^108^, which regulate the differentiation characteristics of MSCs by SRY-box transcription factor 9 (SOX9) or CCAAT enhancer binding protein *β*. As the common products of these pathways, SOX9 interacts with RUNX2 to prevent chondrocytes enter the hypertrophic phase^109^^,^^110^. Consequently, the proper differentiation of MSCs is oriented by the dominance of chondrogenesis mediated by SOX9 over adipogenesis mediated by CCAAT enhancer binding protein *β*^111^.

Moreover, SOX9 and RUNX2 can also act on their regulation pathways in turn to generate the complicate mechanisms for chondrogenesis. The alteration of abundant protein levels above requires the participation of diverse E3 ligases.

### 4.1 E3 ubiquitin ligases regulating TGF-*β*/Smads pathway

Studies have revealed that TGF-*β* promotes chondral proliferation and inhibits hypertrophy *via* phosphorylating Smad3. In this process, COLII and proteoglycan are synthesized to mediate cartilage anabolic processes^112^. Therefore, the hypertrophy of chondrocytes in OA is also verified to be prevented by TGF-*β*/Smad3^113^. Conversely, as one member of Smad families, Smad6 antagonizes the role of TGF-*β* in chondrocytes^114^. TGF*-β* pathway is characterized by the phosphorylation of various Smad family members, during which SMURF2 is highly involved to mediate the ubiquitination of TGF receptors and TGF-activated Smads^107^. In mature chondrocytes, TGF-*β* pathway can be altered by the Smad2/3 deficiency caused by 5-azacytidine-induced SMURF2^115^. Pathologically, elevated SMURF2 is found in cartilage obtained from OA patients. In terms of mechanism, SMURF2 ubiquitinates p-Smad3 to prevent chondrogenesis induced by TGF*β*. Consequently, hypertrophic chondrocytes can produce more COLX and MMP13. SMURF2 overexpression in mouse chondrocytes deteriorates joint cartilage and causes progressive chondral degradation, osteophytes formation and subchondral sclerosis^116^. And decline of SMURF2 can be found in hypertrophic chondrocytes with poor proliferation and differentiation ability^107^. On balance, SMURF2 can positively modulate the maturation of chondrocytes in a variety of manners. As another E3 ligase decreased in cartilage of OA patients, F-box protein 6 is also activated by TGF-*β* to mediate the degradation of MMP13 and MMP14 to restrain the proteolysis of cartilage matrix and alleviate symptoms of OA in mice^113^.

### 4.2 E3 ubiquitin ligases regulating Ihh pathway

Being similar with osteogenesis, IHH secreted by mature chondrocytes bind to its receptor Patched1. Then the releasing of SMO from receptors activates transcription factor GLIs to induce parathyroid hormone related peptide (PTHrP) expression^117^. Accordingly, proliferating but not hypertrophic chondrocytes are the end products and during the process above, WD-40 propeller of the E3 ligase WD-40 repeat-containing SOCS box protein 1 functions on 18-amino-acid loop of type 2 iodothyronine deiodinase to relieve the inhibition effect on PTHrP^118^. Moreover, another E3 ligase ubiquitin protein ligase E3 component *n*-recognin 5 determines the releasing of SMO from Patched-1 receptors to maintain cartilage homeostasis and suppresses metaplasia. Ubiquitin protein ligase E3 component *n*-recognin 5 loss of function in mice induces the serious degradation of articular cartilage, aberrant sesamoid bones and extensive heterotopic tissue metaplasia^119^.

### 4.3 E3 ubiquitin ligases regulating common products of chondrogenic pathways

#### 4.3.1 E3 ubiquitin ligases regulating Sox9

SOX9 is a member of SRY-type high mobility group box family of DNA binding proteins. In MSCs, SOX9 activates the transcription of chondral maker genes such as *Col2a1*, *Col11a2*, *Comp* and *Acan* to establish the direction of differentiation^120^^,^^121^. In chondrocytes, SOX9 inhibits cellular hypertrophy by targeting Wnt/*β*-Catenin pathway and RUNX2^122^. SOX9 also combines the promotor of PTHrP and promotes cell proliferation but not hypertrophy^123^. Therefore, SOX9 is indispensable for various stages of chondrogenesis, and accumulating evidence demonstrated that the degradation of intracellular SOX9 can be mediated by UPS. The coprecipitation between E6-AP and SOX9 has been revealed in nucleus of chondrocytes and the E3 ligase binds to the high mobility group domain of SOX9^124^. Hypertrophic chondrocytes rich in E6-PA lack of SOX9 and the opposite situation is true for the proliferating chondrocytes. With the SOX9 promotion ability, the proteasome inhibitor bortezomib (BTB) is potential to rectify the hypertrophic chondrocytes induced by E6-PA^125^. As a transcription factor, SOX9 also modulates transcription of E3 ligases. The region between exons 4 and 5 of *Wwp2* is mostly recognized by SOX9 to arouse the mono-ubiquitination of Goosecoid, a paired-like homeobox transcription factor required for craniofacial development. Mice lack of WWP2 exhibit malformations of the craniofacial region^126^.

#### 4.3.2 E3 ubiquitin ligases regulating RUNX2

The transcription of chondrogenic genes *Ihh*, *Col10a1* and *Mmp13* can be regulated by RUNX2 directly, the necessity of RUNX2 in hypertrophic chondrocytes has been extensively demonstrated in mice^127^. Subsequently, the effect of RUNX2 ubiquitination on chondrocyte behaviors has also been discovered. WWP2 in chondrocytes contributes to homeostasis of ECM by functioning on RUNX2 directly to improve OA syndromes probably. However, WWP2 deficiency mice do not suffer from craniofacial anomalies under physiological situations^128^. SMURF1 also suppresses RUNX2 indirectly *via* the Smad6 enhancing effect to restrains chondrocyte hypertrophy and maturation^129^.

**Figure 6:**
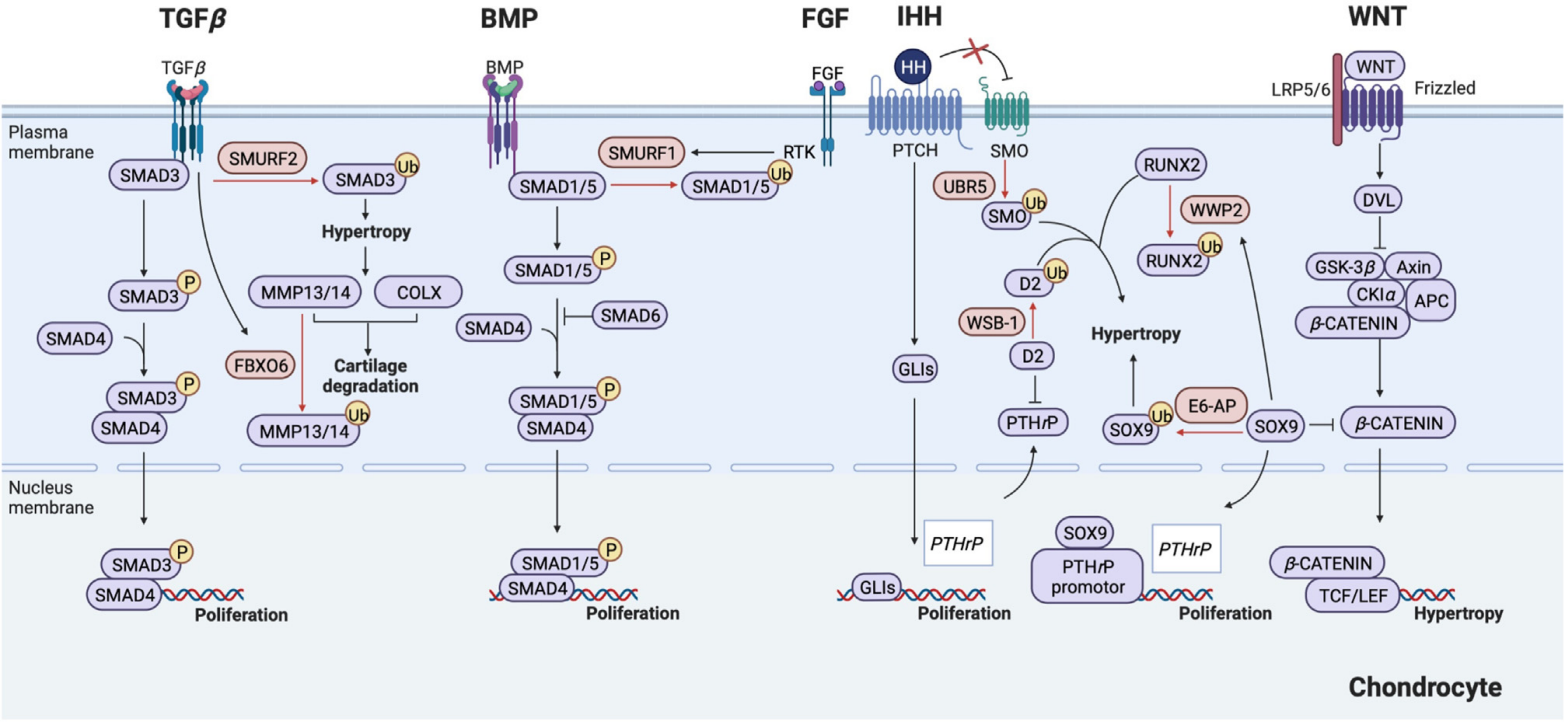
Cartilage behavior relies on the coactions between E3 ubiquitin ligases and key signaling pathways. (A) Transforming growth factor-*β* (TGF-*β*) pathway: TGF-*β*/Smad3-mediated hypertrophic chondrocytes can secrete matrix metalloproteinases (MMPs) for cartilage proteolysis. Smurf2 ubiquitinates both TGF receptor and Smad3. Another E3 ligase F-box protein 6 (FBXO6) induced by TGF-*β*/Smad3 can function on MMPs. (B) BMP pathway: FGF induces Smurf1 to retard BMP pathway through degrading Smad1/5. (C) The crosslink among parathyroid hormone-related peptide (PTHrP), IHH pathway and Wnt/*β*-Catenin pathway: In proliferative chondrocytes, SOX9 promotes PTHrP to restrain RUNX2 induced hypertrophy. On the other hand, SOX9 also promotes E3 ligase WWP2 to ubiquitinate RUNX2. SOX9 also blocks Wnt/*β*-Catenin induced hypertrophy. In hypertrophic chondrocytes, SOX9 is also ubiquitinated by E6-AP and removes the curb on RUNX2 and then IHH pathway is inhibited to limit PTHrP. E3 ligase ubiquitin protein ligase E3 component N-recognin 5 (Urb5) ubiquitinates SMO to inhibit PTHrP and WD-40 repeat-containing SOCS box protein 1 (WSB-1) ubiquitinates type 2 iodothyronine deiodinase (D2) to promote PTHrP.

In conclusion, chondrogenesis is also extensively regulated by the effect of various E3 ligases on different pathways (Fig. 6) and SMURF1 makes the connection between two pathways. For example, SMURF1 mediates the inhibition effect of FGFR3 on BMPRIa/Smad1/5 to prevent chondrocyte differentiation and promote bone dysplasia^130^. Although exploration about the effect of E3 ligase on chondrogenesis probably has great clinical value on cartilage-related diseases, it still remains to be examined because not all chondral pathways are well established to be the targets of UPS.

## 5 The E3 ubiquitin ligases and bone related diseases

Over the last decades, a series of investigations have expounded the involvement of E3 ligases in bone development and homeostasis variously. Bone associated diseases such as metabolic bone diseases, arthritis, bone metastasis of tumors and primary bone tumors, etc., are malignant outcomes of aberrant E3 ligases. As the organ distributed throughout the body, bone tissues are vulnerable to undergo pathological variation, which is also convenient to trouble other organs^131^. In brief, the coming context aims to summarize the role of E3 ligases in bone related diseases and highlight their potential as the therapeutic targets.

### 5.1 E3 ubiquitin ligases regulating metabolic bone diseases

As discussed above, bone turnover is controlled by UPS diversely and associated proteins probably define therapeutic targets for skeletal abnormity. Subsequently, the involvement of E3 ligases in various diseases are summarized in support of this viewpoint. Congenital or acquired factors obstruct normal biochemical state and bone metabolism leading to biochemical metabolic disorders termed as metabolic bone diseases (MBDs). As the third most common endocrine disorders only inferior to diabetes and thyroid diseases, classical MBDs including Paget's disease, sclerosteosis, osteoporosis and rickets, etc., are known to be generally regulated by E3 ligases^132^ (Table 1^36^^,^^51^^,^^62^^,^^76^^,^^126^^,^^133^^,^^134^).

**Table 1:** E3 ligases regulating metabolic bone diseases.

E3 ligase	Substrates	Pathway involved	Major influence of E3 ligases on MBD
SMAD specific E3 ubiquitin protein ligase 1 (SMURF1)	SMAD1; Runt-related transcription factor 2; insulin receptor	Bone morphogenetic protein (BMP) pathway; InsR-osteoprotegerin osteocalcin pathway	Inhibition of osteoblastic SMURF1 promotes bone formation in mouse models of distinct age-related osteoporosis^36^. SMURF1 in mice osteoblasts induces insulin receptor degradation and bone dysplasia^62^.
Ring figure protein 146 (RNF 146)	AXIN1	Fibroblast growth factor 18 (FGF18) pathway	Adipogenesis is enhanced in RNF146^**−/−**^ mouse embryonic fibroblasts. Moreover, RNF146 deficiency in the osteoblast lineage of mice leads to severe osteopenia accompanied with fat accumulation and poor glucose tolerance^51^.
Casitas B-lineage lymphoma proto-oncogene-b (CBL-B)	Receptor activator of nuclear factor kappa-B (NF-*κ*B) ligand (RANKL)	Nuclear factor kappa B (NF-*κ*B) pathway	CBL-B inhibits OCs *via* regulating RANK/NF-*κ*B pathway. Osteopenic phenotype of CBL-B^**−/−**^ mice derives from prosperous bone resorption^76^.
WW domain containing E3 ubiquitin protein ligase 2 (WWP2)	Goosecoid (Gsc)	Goosecoid/SRY-box transcription factor 6 pathway	Mice deficient for WWP2 in OBs develop malformations of the craniofacial region^126^.
Itchy E3 ubiquitin protein ligase (ITCH)	TNF receptor-associated factor 6 (TRAF6)	NF-*κ*B pathway	Clomipramine causes osteoporosis *via* promoting ITCH which is prevented by zoledronic acid in mice^133^.
Cullin 4 B (CUL4B)	Estrogen receptor *α* (ER*α*)	ER*α* pathway	Osteoclastic Nron knockout mice exhibit elevated bone resorption and an osteopenia phenotype. Osteoclastic Nron transgenic mice exhibit totally converse states. In terms of mechanism, the functional motif of Nron interacts with CUL4B to regulate ER*α* stability^134^.

#### 5.1.1 E3 ubiquitin ligases and osteoporosis

Osteoporosis is defined as a systemic skeletal disease characterized by decreased bone mass and degeneration of bone microstructures. Bone fragility and fracture susceptibility are normal complications. The aberrant bone remodeling affects millions of patients each year and has become a serious problem threatening public health. Under the physiological state, bone homeostasis is orchestrated by bone formation and bone resorption spatially and temporally^135^. Osteoporosis will occur if bone resorption is superior to bone formation and basic therapy strategies include healthy diet and outdoor exercise^136^. Moreover, anti-bone resorption or osteogenic promotion medicines are used based on the clinical situations. Severe fracture requires surgical treatments such as internal fixation (plaster fixation, splint fixation), external fixation or artificial joints replacement^137^. Osteoporosis without definite etiology cannot be cured thoroughly.

Drugs used for osteoporosis treatment are majorly divided into anti-resorptive drugs and bone-forming drugs. The long-term effect of anti-resorptive drugs is not fully clear and the side effects are serious. For example, bisphosphonates treatment threatens jawbones and denosumab, the RANKL neutralizing antibody induces necrosis of inferior bone or serious infection. Odanacatib, the specific inhibitor of an osteoclast protease Cathepsin, increases the risk of stroke^138^^,^^139^. For the bone-forming drugs, PTH is expensive and not suitable for the patients with hyperthyroidism or prone to osteosarcoma^139^^,^^140^. Romosozumab approved by U.S. Food and Drug Administration (FDA) recently is the sclerostin neutralizing antibody and enhances WNT pathway by preventing the combination between Sclerostin and LRP5/6^141^. However, the effectiveness of romosozumab is confined to vertebral fractures, which is account for less than 15% of total fractures. Meanwhile, the risk of cardiovascular diseases is also increased^142^. Altogether, exploration of affordable and effective drugs with fewer side effects is the perspective of osteoporosis study. Gradually, numerous publications illustrate the roles of E3 ligases play on bone formation and resorption to provide broader perspective for further study of the mechanisms associated with osteoporosis development.

The concept of repurposing existing drugs for new uses has been granted by FDA since 1997^143^. Although drugs targeted UPS have not been used for clinical therapy of osteoporosis, we list the drugs on the market or not to display the potential effect on bone loss (Table 2^147^^,^^148^^,^^150^, ^151^, ^152^). BTB is the first proteasomal inhibitor and confirmed to improve osteogenesis and restrain bone resorption during multiple myeloma (MM) to protect the skeletal system in mice and humans^144^^,^^145^. The interaction between WNT and LRP5/6 is interrupted by the two cysteine-rich domains of Dickkopf Wnt signaling pathway inhibitor 1 (DKK1). It is worth noting that BTB relieves bone defect by targeting DKK1 in MM patients^146^. The subsequent study confirmed the strengthened WNT/*β*-Catenin pathway in MM patients’ MSCs treated by exogenous BTB^145^. Besides the pathological process of MM, BTB is also found to suppress Smurf–Smad interaction thus exerts pro-osteogenesis functions in OVX mice^147^. BTB also prevents bone resorption of OVX mice by directly inhibiting RANKL-induced osteoclast differentiation^148^. The dual effect of BTB on osteogenesis promotion and bone resorption limitation highlights the enormous potential of BTB to the treatment of osteoporosis. However, wide application of BTB is limited by the off-target effect, drug resistance and toxicity^36^^,^^149^^,^^150^. Clinically, beraprost has been used for alleviating arterial occlusion and the new use of this drug on skeletal system has been studied and approved recently. As the identified inhibitory target of Beraprost, P53 prevents MSCs differentiation through binding to the promotor of *Nedd4* which mediates the degradation of RUNX2. On the other hand, Beraprost also prevents OCs differentiation by reducing the secretion of RANKL. Collectively, although not being confirmed to have therapeutic effect on osteoporosis, the potential effect of Beraprost on bone homeostasis is the dual effects on both promoting osteogenesis and inhibiting bone resorption^151^. As the hormone secreted by the pineal gland, Melatonin has great prospects for treating osteoporosis due to its triple actions on bone formation, bone resorption, and inflammation, which have been identified by clinical research. SMURF1 is the established target of Melatonin in human MSCs to ensure the OBs differentiation. However, the underlying mechanism of Melatonin requires further exploration^150^. Besides osteogenic impairment, osteoporosis is also caused by the side effects of medicines such as clomipramine and the enhancing bone resorption originates from the shortage of ITCH E3 ligase in OCs^133^. E3 ligases are also expected to correct the side effects of other drugs.

**Table 2:** Potential effects of drugs targeting on E3 ligases for osteoporosis treatment.

Drugs	Listed	Current situation	Targets for osteoporosis treatment
Chalcone derivative	No	Pre-clinical research	SMURF1 in OBs^36^
Bortezomib	Yes	Pre-clinical research	SMURF1/SMADs pathway in OBs^147^ RANKL pathway in OCs^148^
Melatonin	Yes	Clinical research	SMURF1 in MSCs^150^
Beraprost	Yes	Pre-clinical research	P53/NEDD4/RUNX2 pathway in Obs^151^
Irisin	No	Pre-clinical research	Focal adhesion kinase/WWP2 pathway in OBs^152^

**Figure 7:**
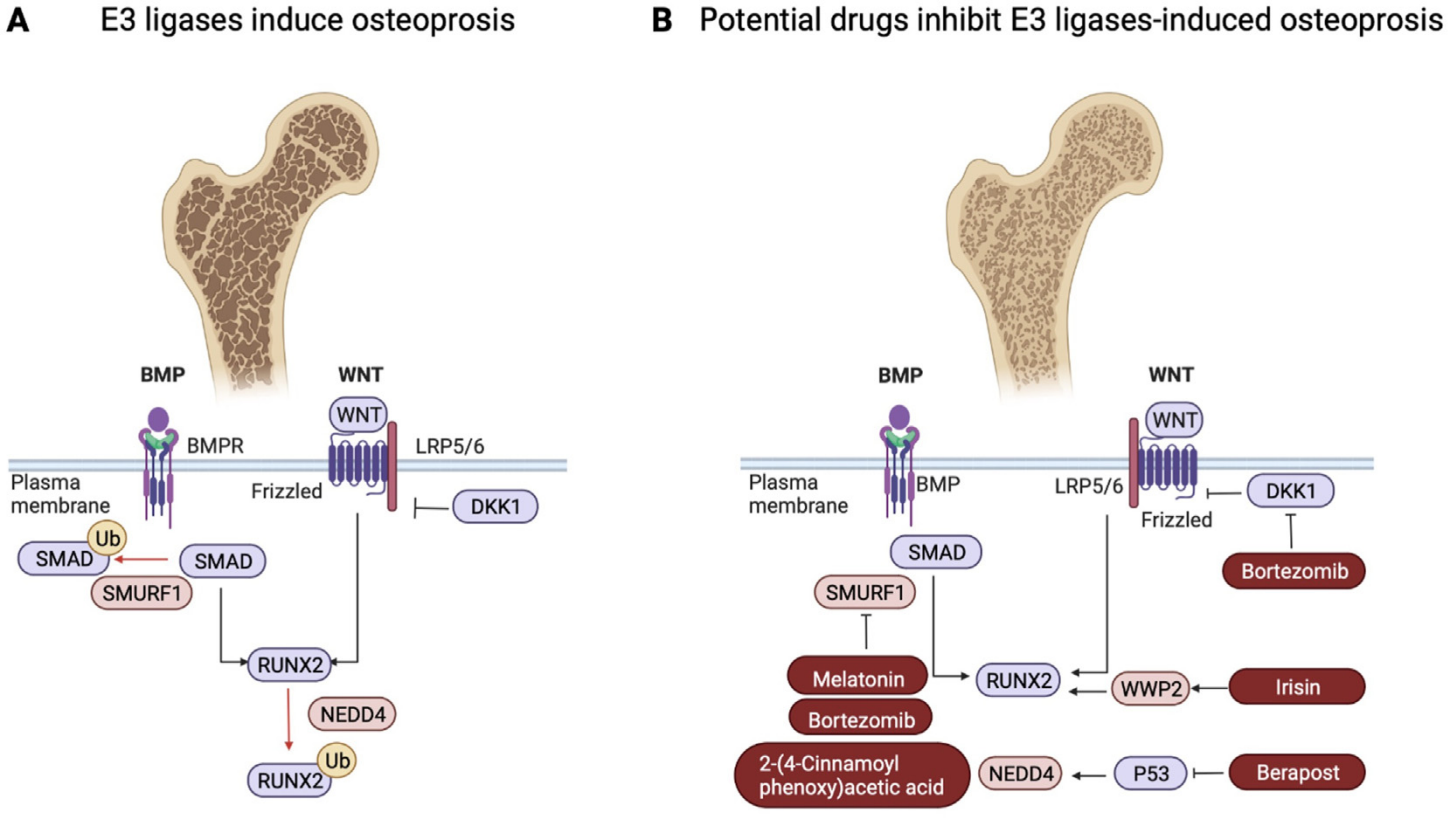
The potential effect on osteoporosis of drugs targeted E3 ligases. (A) Osteoporosis is caused by deficient osteogenesis in response to the aberrant expression of E3 ligases. (B) Drugs listed or not have the potential to rectify osteoporosis by targeting E3 ligases. The effects have been identified by pre-clinical or clinical studies.

As the major E3 ligase in the differentiation processes of osteogenic lineage, treatment of osteoporosis by targeting SMURF1 has been proposed. Being manifested as low SMURF1 activity, mice treated with 2-(4-cinnamoylphenoxy) acetic acid, the chalcone derivative, are free from OVX-induced osteoporosis^36^. In addition to SMURF1, WWPs regulation is also depicted to resist osteoporosis. The muscle-adipose-bone connectivity is strengthened by the existence of irisin, the cleavage protein from the exercise-stimulated skeletal muscle rich in fibronectin type III domain–containing protein 5 (FNDC5) and stimulates focal adhesion kinase to facilitate WWP2 and subsequently activates RUNX1/2. Finally, the skeletal development and white adipose “browning” highlight the therapeutic value of IRISIN in osteoporosis patients with metabolic disorders^152^. However, the evaluation of unlisted drugs requires further researches and trials. The potential effects of these drugs are described in Fig. 7.

Behaviors of OBs and OCs during osteoporosis are also controlled by the alteration of *Smurf1* mRNA level. Both microRNA-195-5 P and long non-coding RNA nuclear-enriched abundant transcript 1 target *Smurf1* to negatively regulate RUNX2 degradation^153^^,^^154^. Another long non-coding RNA *Nron* interacts with E3 ligase CUL4B to mediate bone resorption by the estrogen receptors expressed in OCs^134^. And in OBs, the specific DNA aptamer C3A binds WWP1 to deprive the ubiquitination of RUNX2 to increase deposition of bone matrix^155^. Above all, Nucleic acid-based therapeutics with targeting specificity and long-term effect provide novel strategies targeted E3 ligases for osteoporosis treatment.

#### 5.1.2 E3 ubiquitin ligases and Paget's disease

Paget's disease, also called deformans osteitis and the morbidity is mainly found in patients over 40 years old. The clinical syndromes are bone deformation, bone pain, fracture and even the deafness. The coupling between robust activation of OBs and OCs accelerate the bone resorption accompanied with enostosis and irregular osteogenesis^156^. So local bones, such as pelvis, skull, spine and leg bones are enlarged but with low bone density. Studies established recently demonstrated that mutation of Valosin-containing protein at A232E harbored enhancing ATPase activity and led to the Paget's disease of bone-like syndrome^157^. In the process above, I*κβ*1 ubiquitinated by SCF*β*-TrCP is enhanced to derepress NF-*κ*B pathway in the hyperactivated OCs and elevated SMURF1 in OBs impairs BMP/Smad pathway^158^. Therefore, UPS mediates the dual modulations of OBs and OCs activity and provide novel therapeutic perspective of Paget's disease.

#### 5.1.3 E3 ubiquitin ligases and osteopetrosis

Normal OCs and OBs collaborate to generate more bone matrix in the stress area and less matrix in the stress-free area. In the osteopetrosis patients, H^+^ cannot be produced when OCs suffer from the mutation of carbonic anhydrase 2^159^ and the impairment of bone resorption results from disabled OCs. Although having the opposite mechanism with osteoporosis, fracture is also prone to appear during osteopetrosis^160^. Normally, osteopetrosis associated transmembrane protein 1 might function as an E3 ligase to regulate Wnt signaling cascades. Mutation of osteopetrosis associated transmembrane protein 1 is found to cause severe autosomal recessive osteopetrosis in both mice and humans^161^.

#### 5.1.4 E3 ubiquitin ligases and Rickets

Vitamin D promotes the resorption and utilization of calcium and phosphorus. Proper development of bones depends highly on the calcium and phosphorus deposition on the growing areas of bones^162^. Rickets is a malnourished syndrome occurs during the infancy. The abnormal skeleton is induced by the hyperactivity of the parathyroid gland and lack of vitamin D induces the metabolic disorders of calcium and phosphorus^163^. As is shown before, osteogenic alteration brought by PTH should be attributed to the effect of E3 ligases on OBs or OCs. Therefore, although the direct effect of E3 ligases on Rickets is not published, the presenting discoveries supply promising concept for the therapy of Rickets on UPS.

### 5.2 E3 ubiquitin ligases regulating tumor bone metastasis

Breast cancer (BC) and prostate cancer (PC) are two leading causes of cancer-associated morbidity and mortality in females or males respectively^164^. More than 70% of advanced BC or PC patients undergo bone metastasis and skeletal-relate-events^165^, ^166^, ^167^, which include pathological fractures, spinal cord compression and the adverse reactions of cancer treatment, such as palliative radiation and surgery interventions, to the bone. Multiple osteolytic lesions are commonly diagnosed as the bone metastasis of BC^167^, while osteogenic lesions are common in that of PC^168^. Due to different clinical manifestations between the bone metastasis of BC and PC, scientists strive to explore potential molecular mechanisms by means of primary tumor cells. Existing evidences demonstrate that specific interactions between bone microenvironment (BME) and primary tumor cells can generate therapeutic resistance by promoting excessive invasion and growth of tumor tissues^169^^,^^170^. Of note, E3 ligases are modulators for degrading pivotal molecules involved in the communication between the primary tumor cells and BME. Compared with BCs patients with normal WWP1, worse prognosis is the obvious feature of patients with lower WWP1 level^171^, ^172^, ^173^. It can be interpreted that the absence of WWP1 promotes BC cells migrating to the bone marrow by inhibiting CXCR4 degradation^173^. Recently, beta-transducin repeat containing E3 ubiquitin protein ligase in BCs ubiquitinates ULK1 to accumulate damaged mitochondria. Accordingly, enhanced bone resorption competence from dynamic OCs profits from the activated NLR family pyrin domain containing 3 (NLRP3) inflammasome and secretion of osteolytic factors Interleukin-6 (IL-6) and IL-1*β*. These abnormal soluble cytokines directly destruct bone tissues and ultimately facilitate the progression of tumor cell bone metastasis^174^. More recently, it was investigated that E3 ligase COP1 was an immunotherapy target in triple-negative breast cancer (TNBC)^175^. Notably, brain, liver and brain metastasis are more common in TNBC patients^176^. While non-TNBC patients, account for 75% of total BC cases, are more likely to develop bone metastasis^169^^,^^175^^,^^177^^,^^178^. Some key E3 ligases mediate the interaction between tumor cells and OBs or OCs in non-TNBC needs further explored. It has been revealed that the process of osteolytic bone metastasis in BC is a destructive feedback loop. Tumor cells produce RANKL to function on OCs and facilitate the cellular differentiation and activation. Then the osteolysis enhances the release and activation of growth factors to promote tumor growth^179^. However, the participation of E3 ligases in this destructive “vicious circle” remains unknown and provides broad prospects for the following research.

PC cells can disequilibrate bone homeostasis by promoting the secretion of osteoblastic factors such as BMPs^180^, ^181^, ^182^, ^183^, TGF-*β*^184^, and Endothelin-1^185^, etc., and building the specific niche BME suitable for the colonization and proliferation responses of tumor cells^186^. An experimental study has shown that PC cells tend to enter dormant state for adaptation when they arrive in the BME^187^. Intriguingly, Wnt5a secreted from OBs can mediate the dormancy of PC cells in a reversible manner. The underlying principle is the enhancing expression of Siah E3 ubiquitin protein ligase 2 which promotes the degradation of *β*-Catenin and decreases the expression of downstream target genes. In order to induce the dormancy of PCs in BME, WNT5a ubiquitinated by Siah E3 ubiquitin protein ligase 2 is potential to play vital roles although the clinical significance of which has not been established.

On account of possessing E3 ligase activity, the expression and potential functional association of tripartite motif (TRIM) family proteins has also been deciphered in bone metastatic of PC by Offermann et al^188^*.* This study systematically clarified that the signaling pathways, including PI3K/AKT, TNF-*α*, TGF-*β* and HIF-1 significantly correlated with differently expressed TRIM proteins, such as TRIM5, TRIM10, and TRIM42, etc. Moreover, interaction and adhesion between proteoglycans and ECM receptors are the classical cellular processes associated with PC cells and BME. Notably, E3 ligases may have a major impact on mediating the degradation of crucial substrates in metastatic PC. This study provides a guideline for further research around functions of specific TRIM or other E3 ligases on this detrimental process.

Mechanistically, all the present therapies around tumor bone metastasis dedicate to inhibit tumorigenesis and bone resorption. The E3 ligases discovered in tumor bone metastasis have not been identified in OCs behaviors. It is highly necessary to study the role of E3 ligases regulating the activity of both tumor cells and OCs to reveal the potential therapeutical effects of E3 ligases involved.

### 5.3 E3 ubiquitin ligases regulating primary malignant tumors originating from bones

#### 5.3.1 E3 ubiquitin ligases and osteosarcoma

Osteosarcoma is the most frequent solid tumor in bones and often occurs in children or young adults. Patients undergo prominent symptoms because tumor tissues erode and dissolute the cortical bone to induce the pain of lesion area. The predominating direction of UPS research on osteosarcoma cells (OSCs) is the regulation of cellular growth (Table 3^174^^,^^184^^,^^185^^,^^192^, ^193^, ^194^). RING finger LIM domain-binding protein can degrade Stathmin, the oncopr-otein highly expressed in a wide variety of human malignancies including osteosarcomas, to abolish cellular division and proliferation^189^. In OSCs, as the crucial member of Cullin-RING E3 ligase, Cullin 4 B (CUL4B) is verified to bind DNA damage binding protein 1 (DDB1) and CUL4-associated factor 13 to constitute E3 complex in human OSCs which degrades PTEN, the tumor suppressor, and promotes cell growth. Moreover, the 3′-UTR of *CUL4B* is targeted by microRNA-300 which sustains the stability of PTEN^190^. CUL4B also forms an E3 ligase complex with RING-box 1, DDB1 and CUL4-associated factor 11, which specifically ubiquitinates a cyclin-dependent kinases inhibitor—p21^Cip1^. Cell proliferation retarded by arresting cells at S phase can be rescued^191^.

**Table 3:** E3 ligases regulating bone tumors or tumor bone metastasis.

E3 ligase	Substrates	Pathway involved	Major influence of E3 ligases on bone tumors or tumor bone metastasis
Beta-transducin repeat containing E3 ubiquitin protein ligase (BTRC)	Phosphorylated Unc-51 like autophagy activating kinase 1 (P-ULK1)	Mitogen-activated protein kinase kinase 2/MAP kinase kinase 1/mitogen-activated protein kinase **(**MAP2K/MEK/MAPK1/3) Pathway	BTRC induces the degradation of P-ULK1 to promote breast cancer bone metastasis^174^.
CUL4B	Phosphatase and tensin homolog (PTEN); p21^Cip1^		CUL4B binds to DNA damage binding protein 1 (DDB1) and DNA damage binding protein 1 and CUL4-associated factor 13 (DCAF13) to form complex which can degrade PTEN to promote cell growth of Osteosarcoma cells (OSCs)^184^. CUL4B combines with RING-box 1, DDB1 and DCAF11 to degrade p21^Cip1^ and facilitate cell proliferation of OSCs^185^.
Developmentally downregulated 4 (NEDD4-1	AKT	Phosphatidylinositol-3-kinase/Protein kinase B (PI3K/AKT) pathway	NEDD4-1 promotes the sensitivity of human myeloma cell line to Bortezomib through PTEN/PI3K/AKT pathway^192^.
Human ubiquitin–protein ligases 1 (HUWE1)	MYC		HUWE1 depletion and lenalidomide have synergistic effect on multiple myeloma (MM) treatment both *in vitro* and *in vivo* models^193^.
Denticleless E3 ubiquitin protein ligase (DTL)	Inhibitor of NF-*κ*B	NF-*κ*B pathway	DTL is highly expressed in MM cells and mononuclear cells isolated from 34 MM patients^194^.

However, Haydon et al*.*^195^ put forward the alteration from osteosarcomas to physiological osteogenesis. Compared to conventional chemotherapy, terminal differentiation of OSCs may be a promising treatment for osteosarcoma^195^. Even so, the UPS dependency of OSCs benign differentiation was assumed almost one decade later. During osteogenesis, OBs differentiation benefits from all-trans retinoic acid (ATRA), the derivative of vitamin A which can combine retinoic acid receptor *α*. Although not being established clinically, OSCs are demonstrated to differentiate into OBs in response to ATRA by studies *in vitro* and *vivo*. As the E3 ligase, human homolog of murine double minute-2 is also an oncogene over-expressed in different types of malignancies, such as OSCs. Consequently, stable retinoic acid receptor *α* is eliminated to prevent the OBs differentiation. The switch of osteosarcoma into normal osteogenesis broadens the avenue of therapeutic investigation around malignant tumors^196^.

#### 5.3.2 E3 ubiquitin ligases and multiple myeloma

As the second most common hematologic malignancy, the incurable multiple myeloma (MM) is the malignant disease derives from the runaway growth of plasma cells in the bone marrow. Overproduction of nonfunctional intact immunoglobulins or immunoglobulin chains interact with other bone marrow cells to induce problems such as anemia, bone lesions, infections, hypercalcemia, renal failure, fatigue and pain^197^. As the highly conserved serine–threonine kinase, AKT is plentiful in MMs and motivates cellular functions by recognizing a series of substrates. And it is well known that the activity of tumor cells relies on the frequent alteration between PI3K/AKT and PTEN. pAKT-Ser473 is identified as the degradation site of NEDD4-1, the certified tumor-suppressing E3 ligase in multiple malignancies^192^. However, on the contrary, the oncogene *c-MYC* is highly expressed under the treatment of human ubiquitin-protein ligases 1, another large (482 kDa) HECT-domain E3 ligase which activates transcription of MYC through K63-linked ubiquitination but inhibits MYC through K48-linked ubiquitination in different cell types or context^193^. Similarly, denticleless E3 ubiquitin protein ligase in MM cells sustains cellular functions *via* NF-*κ*B, the factor which is crucial to proliferation and drug-fast capability of MM cells^194^ (Table 3).

Verification of the roles of listed drugs functioning on multiple myeloma has already been widely published. The immunomodulatory drug (IMiD) Thalidomide and its derivatives lenalidomide and pomalidomide are therapeutic agents used in the treatment of multiple myeloma^198^. In MM cells, as the highly effective medicine, Lenalidomide binds DDB1 and Cereblon (CRBN) which generate CRL4^CRBN^ E3 ligase (CRL4) with CUL4 and regulator of Cullins 1. Essential transcription factors IKAROS family zinc finger 1 (IKZF1) and IKZF3 in multiple myeloma are targets of CRL4-CRBN E3 ligase^199^. The mutation of IKZF1, CRBN or CUL4B in MM cell lines can generate drug resistance. And MM patients are also found to undergo these gene mutations. After IMiD treatment, the mutations occur in E3 ligase but not in these transcription factors are reversible^200^. Differently, thalidomide binds to CRBN directly and interacts with DDB1 indirectly^201^. Although several neo-substrates have previously been reported, the diverse pharmacological activities of IMiD drugs prompt us to explore new substrates of CRL4^CRBN^. Unlike other UPS members, CUL4-based E3 ligases require an intermediary factor for recognizing some substrates indirectly^202^^,^^203^. AT-rich interaction domain 2, a component of the polybromo-associated BAF chromatin-remodeling complex, is a Pomalidomide-induced neo substrate that is important in the expression of plentiful pomalidomide target genes, including *MYC*. However, lenalidomide has no effect. As a result, pomalidomide offers considerable clinical benefits to patients with lenalidomide-resistant multiple myeloma^204^.

Additionally, being manifested as the postponement effect on tumor growth which brings about the longer period without progression and more survive time for patients, the proteasome inhibitor (PI) BTB is an effective drug for MM therapy^205^. However, MM patients with poor prognosis and even the recurrence can be accused to the drug resistance, which is caused by the reversible binding of BTB to 20 S proteasomes. Huang et al. reported that the sensitivity of MM under BTB intervention was abolished by the absence of NEDD4-1 and the reaction above was reversible if supplementary NEDD4-1 was added^192^.

Compared with the therapeutic effect of BTB on MM, the second-generation PI Carfilzomib has higher efficacy and tolerability due to the irreversible binding to 20S proteasomes. It is used for patients who have received at least two drugs for MM^206^. The oral ixazomib is the third PI approved by FDA. Ixazomib is used in combination with lenalidomide and dexamethasone for the treatment of MM in patients who have received at least one line of previous therapy^207^.

Clinically, coadministration of lenalidomide, bortezomib and monoclonal antibodies are referred to as triplet regimens, which are used in the early stage of MM. Switching drugs to a further generation agent is adopted when patients relapse and are refractory to existing regimens^208^. In Table 4^201^^,^^204^, ^205^, ^206^, ^207^^,^^209^, ^210^, ^211^, drugs targeting UPS have been summarized and illustrated in detail. Altogether, therapeutical strategies should be made according to the individual patient. Meanwhile, exploration of novel agents is also required to supply optimal treatments during various periods of MM.

**Table 4:** Current situation of clinical drugs for multiple myeloma treatment.

Drugs	Targets	Advantages or disadvantages
Lenalidomide	Cereblon (CRBN), DDB1	1. Compared with thalidomide, the efficacy is better, and the toxicity is lower^199^. 2. Myelosuppression effect^209^.
Thalidomide	CRBN	1. Unstable combination with CRBN^201^. 2. Teratogenic effect^210^.
Pomalidomide	AT-rich interaction domain 2	Pomalidomide is only limited to the treatment of MM^204^.
Boterzomib	*β*1 and *β*5-subunit of 20 S proteasome	Off-target effect, drug resistance and toxicity^205^.
Carfilzomib	*β*5-subunit of 20 S proteasome	1. Compared with boterzomib, the efficacy and tolerability are better^206^. 2. Cardiotoxicity^211^.
Ixazomib	*β*5-subunit of 20 S proteasome	Oral drugs provide convenient therapy^207^.

### 5.4 E3 ubiquitin ligases regulating arthritis

Inflammatory disorders occur in joints and surrounding tissues are collectively known as arthritis. Over 100 million Chinese are plagued by arthritis in varying degrees such as the redness, swelling, heat, pain, deformity and handicap of joints^212^. Consequently, low life quality and shortened life span emerge in patients. Arthritis can be classified by the probable etiology and the most common form are osteoarthritis, rheumatoid arthritis and ankylosing spondylitis. The effect of E3 ligase on arthritis is shown in Table 5^18^^,^^116^^,^^128^^,^^189^^,^^213^, ^214^, ^215^, ^216^, ^217^, ^218^, ^219^, ^220^, ^221^.

**Table 5:** E3 ligases regulating arthritis.

E3 ligase	Substrates	Pathway involved	Major influence of E3 ligases on arthritis
CBL-B	Phospholipase C*γ*-1 (PLC*γ*-1)		Loss of CBL-B ensures T cell tolerance and excessive auto immunity arthritis. Phosphorylation of PLC*γ*-1 rescues the disabled T cells. Therefore, CBL-B in T cells prevents the occurrence of autoimmunity arthritis^18^.
SMURF2	SMAD3	Transforming growth factor/SMAD3 pathway	SMURF2 is up-regulated in osteo arthritis (OA) patients. SMURF2-transgenic mice in chondrocytes suffer from OA-like phenotype induced by p-SMAD3 degradation^116^.
WWP2	ADAM Metallopeptidase with Thrombospondin Type 1 Motif 5 (Adamts5)	Runx2–Adamts5 pathway	Mice lacking WWP2 in chondrocytes exhibit aggravated spontaneous and surgically induced OA^128^.
Rlim (RNF12)	Stathmin		RNF12 is a novel regulator of STATHMIN in human osteosarcomas^189^.
HMG-CoA reductase degradation protein 1 (Hrd1)	P53	The endoplasmic reticulum-associated degradation pathway	*Hrd1* knockdown suppresses the proliferation of rheumatoid synovial cells to reduce the severity of rheumatoid arthritis (RA). Therefore, HRD1 inhibition is a potentially effective treatment for RA^213^.
Von Hippel-Lindau (VHL)	Hypoxia-inducible factor-1*α* (HIF-1*α*)	HIF-1*α* pathway	HIF-1*α* can be up-regulated in the synovium of RA patients and VHL-mediated degradation of HIF-1*α* can prevent RA^214^.
Cullin1	Interleukin-8 (IL-8)		Cullin1 in T cells inhibits IL-8 and probably improves RA syndromes^215^.
ITCH	Jagged canonical Notch ligand 1 (JAG-1); NF-*κ*B	NOTCH Pathway; NF-*κ*B pathway	ITCH improves LPS-induced human chondrocytes by ubiquitinating JAG-1^216^. Itch inhibits post-traumatic OA progression by preventing macrophage polarization and pro-inflammatory effect^217^.
F-Box and WD repeat domain containing 7 (FBXW7)	Mitogen-activated protein kinase kinase 7 (MKK7)	JNK pathway	FBXW7 deletion in chondrocytes induces chondrocyte senescence and accelerates cartilage catabolism in mice^218^.
Ubiquitin-fold modifier 1-specific ligase 1 (UFL1)	IL-1*β*	NF-*κ*B pathway	UFL1 level is down-regulated in OA cartilage and UFL1 exerts protective effect on IL-1*β*-induced chondrocytes. UFL1 probably inhibits osteoarthritis^219^.
SMURF2	Pentraxin 3 (PTX3)	FGF pathway	SMURF2 in mesenchymal stem cells (MSCs) from ankylosing spondylitis patients degrades PTX3 to facilitate FGF pathway and angiogenesis^220^^.^
Tripartite motif containing 65 (TRIM65)	NLR family pyrin domain containing 3 (NLRP3)		TRIM65 deficiency aggravates lipopolysaccharide-induced systemic inflammation and Monosodium urate- induced peritonitis and gouty arthritis *in vivo*^221^.

#### 5.4.1 E3 ubiquitin ligases and rheumatoid arthritis

Rheumatoid arthritis (RA) is the chronic inflammatory joint disease affect facet joints on hands or feet symmetrically. The effusion in the inflammatory synovium leads to pain, stiffness and swelling of joints. Current therapeutic regimens around RA are symptomatic treatment and disease modifying management (DMARDs). Oral nonsteroid ant-inflammatory drugs (NSAIDs) and glucocorticoids (GCs) can alleviate the inflammatory syndromes and arthralgia. They have a rapid response but serious side effects and do not fundamentally cure RA. By contrast, although being slow-acting drugs, DMARDs fundamentally delay RA development by suppressing immunity and preventing joint degeneration. Gastrointestinal stimulation, hepatic injury, leukopenia and rash etc. are the most common side effects of DMARDs^222^^,^^223^.

E3 ligase Von Hippel-Lindau (VHL) mediates the degradation of hypoxia-inducible factor-1*α*, which is extensively distributed in the hypoxia areas of RA, to maintain homeostasis by creating normal conditions. RA symptoms may be improved by attenuating the effect of VHL^224^. Pathologically, RA patients suffer from excessive growth of synovial cells^225^. As a RING-type ligase E3, synoviolin is abundantly found in synovial cells of RA patients. It contributes to the proliferation of synoviocytes and prevents apoptosis induced by ER stress *via* ER-associated degradation^213^. Therefore, synoviolin inhibitors LS-101 and LS-102 are proposed to be optimized to develop novel therapy for RA^213^^,^^214^.

In addition to skeleton system, significant correlation between RA and E3 ligase has also been confirmed in immunity system. Culin1 in immunological tissues aggravates susceptibility to RA *via* a single nucleotide polymorphism in intron 3 of the *CUL1* gene to alter transcriptional efficiency. The Chemokine IL-8 is positively regulated and the mechanism is not completely known. SiRNA imposed on Culin1 can alleviate RA by restricting IL-8 production, which directs inflammatory cells to lesion areas in RA^215^. Loss function of CBL-B in T cells results in the weakened tolerance to their own antigens *via* augmenting phosphorylation of Phospholipase C*γ*-1. Therefore, RA in CBL-B^−/−^ mice is more easily to be evoked by COLII even though without mycobacterial adjuvants^226^. E3 ligases in T cells provide novel targets for the vaccine design in RA, the autoimmune diseases.

#### 5.4.2 E3 ubiquitin ligases and osteoarthritis

Being mainly featured with the evident joint pain and stiffness, senescence-associated osteoarthritis (OA) is the most frequent degenerative joint disease accompanied with impairment of articular cartilage and cartilage matrix. Prevalence of OA is mainly concentrated in old people and the disability rate is only secondary to cardiovascular diseases. Although it remains unclear about the exact etiology, the superiority of cartilage degradation to synthesis is identified as the principal element underlies OA^227^.

In the early or medium stage of OA, oral drugs alleviate the inflammatory syndromes and arthralgia. However, NSAIDs are detrimental to gastrointestinal tract^228^. Furthermore, as the inherent constituent of cartilage and synovia, hyaluronic acid (HA) provides joints with higher security. Intra-articular administration of HA relieves synovial inflammation, cartilage destruction and enhances joint function. Intra-articular injection of GCs is performed when other treatments are invalid or the acute exacerbation of inflammation emerges. Meanwhile, GCs with serious side effects should be used with caution^229^. In a word, although improving syndromes of OA, current drugs failed to prevent the course of the disease.

E3 ligases have been majorly reported to improve OA syndromes probably *via* influencing chondrocytes and cartilage state. Vital effect of Jagged Canonical Notch ligand 1 (JAG1)–Notch1 pathway has been highlighted in OA cartilage tissues and identified as the substrates of ITCH. The WW-PPXY motif of the E3 ligase executes degradation on JAG1 *via* K48 ubiquitination. In parallel, the anti-OA effect of ITCH was also reflected in LPS Lipopolysaccharide-induced chondrocyte damage, which can be reversed by JAG-1^216^. Moreover, ITCH has the intrinsic anti-inflammation effect and *LysM-Cre;* Itch^fl/fl^ mice lack of ITCH specially in macrophages are found to promote polarization of macrophages and post-traumatic OA joints^217^. Therefore, as the ITCH inhibitor, clomipramine should be prescribed with caution to avoid accelerating OA and even the bone loss. Future research should be performed on the pharmacologic inhibition of ITCH degradation for the new approach of OA treatment. With the increasing burden on mice articular cartilage, F-box and WD repeat domain containing 7 E3 ligase is decreased at the transcription level and mitogen-activated protein kinase kinase 7 is preserved to stimulate JNK-mediated chondrocyte senescence^218^.

Interestingly, although E3 ligase WWP2 has no relationship with OA, the intronic miR-140 presents in the WW domain of WWP2 and decreases the expression of genes known to play detrimental roles in OA cartilage. Other proteins except WWP2 act as the regulators of miR-140 expression to restrain cartilage lesions. It is reported at the first time that there is a differential expression between miR-140 and its host gene^230^. However initially, the co-expression between microRNA and their host genes makes us believe the source of intronic microRNAs governance. Gradually, more study showed that being independent of their host genes, some intronic miRNAs have their own promoters and their behaviors are out of the command from the host genes.

Compared with RA, OA is also the chronic inflammation but mainly implicates heavily operated or burdened joints and appears as the degradation of articular cartilage^227^. The E3 ligase ubiquitin-fold modifier 1-specific ligase 1 (UFL1) is possible to rectify OA *via* resisting NF-*κ*B signaling pathway in chondrocytes. Consequently, the pro-inflammatory viability imposed on chondrocytes exposed to IL-1*β* is safeguarded^219^. UFL1 in chondrocytes is less present in OA patients. After being exposed to IL-1*β*, inflammatory pathways and factors are activated in chondrocytes. Matrix degradation is also promoted by more synthesis of MMPs and ADAM metallopeptidase with thrombospondin type 1 motif. The research applied on OA *in vitro* shows that all the adverse reactions above are abolished by the ubiquitination of IL-1*β* derived from ULF1^219^.

Altogether, there is no specific medicine for OA and the present treatments aim to improve symptoms. The influence of E3 ligases on inflammation provides novel therapeutic strategy for arthritis treatment.

#### 5.4.3 E3 ubiquitin ligases and ankylosing spondylitis

As another chronic and inflammatory arthritis, ankylosing spondylitis (AS) affects the joints of pelvic bones and spines to cause pain and stiffness at lesion areas. The etiology of AS is largely dependent on pathological angiogenesis characterized by dense but permeable vasculature at bone-cartilage interface and subchondral bone marrow. The inflammatory factors from the circulation blood are abundantly transported and penetrate to intensify the lesion areas^231^. Physiologically, angiogenic factors derived from MSCs sustain the normalization of endothelial behaviors, which can be aberrantly enhanced by superfluous angiogenic factors. Moreover, it is worth noting that MSCs from AS patients are discovered to be rich in SMURF2, which ubiquitinates pentraxin 3 to remove its high affinity with FGF2. Then crucial angiogenic factors are freed to generate abundant immature angiogenesis, which in turn exacerbates the syndrome of AS^220^. The restraint of excessive angiogenesis may be the correct option for AS rather than just blocking Smurf2. It is necessary to develop drugs around EGF-related pathways.

#### 5.4.4 E3 ubiquitin ligases regulating inflammatory arthritis in the mode of “across organ”

As the organ distributed throughout the body, inflammatory lesions occur in skeletal system can be induced by either internal disorders of bone homeostasis or abnormity of external organs such as the liver, vessels and immunity system^232^, ^233^, ^234^. In macrophages, E3 ligase TRIM65 directly binds NLRP3 inflammasome to promote ubiquitination at the Lys48 and Lys63 sites. TRIM65 deficiency macrophages undergo NLRP3 recovery and regain the inflammatory ability. The reaction above can aggravate the gouty arthritis in mice performed by intraperitoneal injection of mono-sodium urate^221^. However, the interaction between UPS and the vasculature during bone metabolism is almost still unexplored. The recently proposed “bone–vessel axis” illuminates the angiogenic regulation is able to change bone homeostasis in the mode of “across organ”. Ma et al.^220^ unprecedently proposed the interaction between UPS in MSCs and endothelial functions during inflammatory arthritis. Although almost being in a blank field, the involvement of UPS in the established bridge between bone and vessels broadened our vision around UPS influence on other organs-induced bone disorders such as RA, diabetic osteoporosis, and hepatic osteodystrophy, etc.

## 6 Conclusions

On account of the unknown pathogenies, existent treatment of MBDs or arthritis is restricted to improve symptoms rather than preventing the courses. And lesions of other organs may result from the adverse reactions. As to tumor bone metastasis and tumors originating from bones, both chemotherapy and radiotherapy mainly induce myelosuppression, gastrointestinal reactions, skin or oral problems. Besides, chemotherapy also causes peripheral neuropathy, cardiotoxicity, hepatic and renal dysfunctions etc. However, chemotherapy and radiotherapy are still the preferred treatment for advanced tumors^235^.

Therapeutic actuality forces us to search for novel therapeutic mechanisms. Being equipped with multiple E3 ligases, the ubiquitination–proteasome system dedicates to sustain the homeostasis of proteins. Proteolysis of unnecessary proteins is implemented specially by UPS in a manner of energy dissipation. Therefore, intracellular UPS is an important regulator of both physiological and pathological processes. PIs function on UPS-dependent pathways indiscriminately are not conducive to normal cells. Therefore, the advantages of drugs targeting E3 ligases specifically are prominent. Screening inhibitors of these small molecules or exploiting targeted antibodies may be determined by the specific structure of E3 ligase^236^. Physiologically, some E3 ligases are poorly expressed in normal tissues^237^. Therefore, drugs recognizing E3 ligase particularly may impact normal cells less. Altogether, targeting E3 ligases specifically probably enhances therapeutical effects and avoids side effects to the greatest extent.

Among the numerous E3 ligases, structure-based drug design contributes to discover more E3 ligase inhibitors or agonists with high specificity^238^. Available technologies, such as high throughput screening and computer virtual screening fulfill to screen more small molecular regulators of E3 ligases^239^. Further deciphering protein crystal structure of E3 ligases and the mechanism on substrates are beneficial to discover more potential combining sites for drugs^240^. However, small molecular inhibitors are potential to suffer from off target and unexpected side effects. Some E3 ligases relies on the protein–protein interactions derived from complexes and the small molecular inhibitors failed to capture the changing spatial conformation of proteins^241^. Not only that, E3 ligase inhibitors may not affect bone system efficiently because of their different target proteins. Therefore, people are facing the huge challenge of medicine development around targeting E3 ligases. As the bridge between ubiquitin and target substrates, the influence of E3 ligases on bone metastasis, chondrogenesis and tumorigenesis are being deciphered gradually by increasing studies. In addition to the skeletal system, the superiority of UPS has been made in the regulation of other systems such as vasculature to influence osteogenesis likewise. Therefore, based on these theories, therapies around UPS have been widely applied in clinical practice. Although the development of drugs targeting E3 ligase has been focused on tumors resistance for a long time^242^, the crucial roles of E3 ligases have already been published in neurodegenerative diseases, diabetes, autoimmune diseases and inflammation, etc. by extensive studies^243^, ^244^, ^245^.

**Figure 8:**
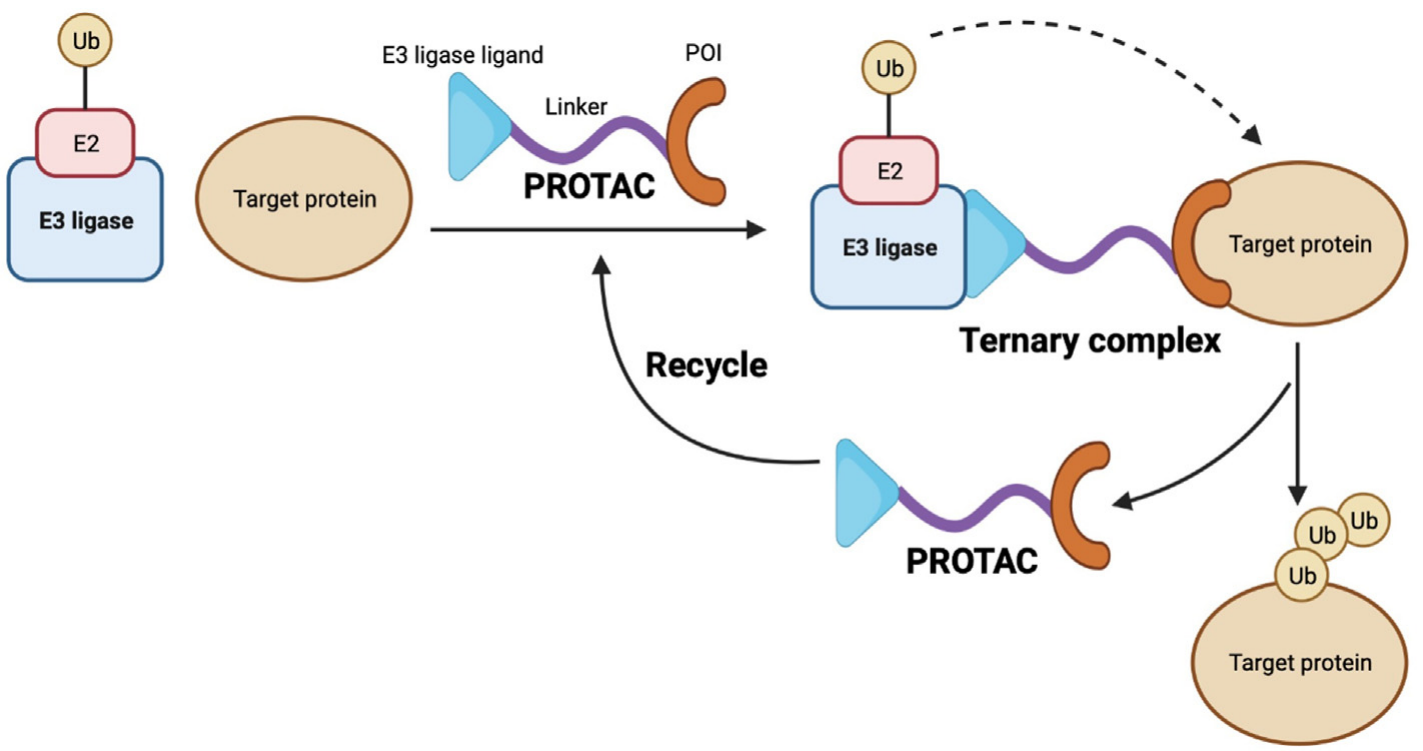
Schematic shows the basic principles of proteolysis targeting chimeras (PROTAC). The E3 ligase binds to Ubs conjugated enzyme E2. Being equipped with E3 ligase ligand, target protein ligand and linker structure, PROTAC is the bridge between E3 ligase and target protein to mediate the ubiquitination of target proteins. Finally, PROTAC molecules are recycled for the future use and ubiquitinated proteins are degraded.

Recently, PROTAC (proteolysis targeting chimeras) design is based on the UPS principle and responsible to degrade the target proteins. PROTAC consists of an E3 ligase ligand, a target protein ligand (POI) and a “linker” structure. Eventually, the triplet PROTAC is generated in the active form. After PROTAC molecules entered into cells, POI can combine with target proteins specifically. The other end recruits E3 ligase to form the ternary complex POI-PROTAC-E3 ligase. The E3 ligase mediates the ubiquitination of POI to dissociate ternary complex. Ubiquitination of target proteins can be processed instantaneously by the transient formation of ternary complex and PROTAC is recycled for future uses (Fig. 8).

As the catalysis-like reaction, the requirement of PROTAC drugs on pharmacodynamic time, strength and dose is very low to ensure the safety and efficacy. Ligand design based on tissue specificity recognition of E3 ligases contributes to the selective degradation of target proteins. Traditional small molecular drugs occupy key sites of target proteins to produce pharmaceutical effect. PROTAC drugs are independent of active sites and combine any sites of target proteins theoretically. Being not recognized and targeted at indicated sites, 80% proteins cannot be modulated by pre-existing drugs. Theoretically, protein degradation occurs when any sites of them are combined by novel drugs. Therefore, PROTAC is highly potential to function on proteins or even diseases not affected by current drugs. Moreover, the compatibility between PROTACs and mutated proteins overcome the drug resistance^246^. However, some disadvantages still exist. Huge molecules of PROTAC inducing the permeability problem and clinical safety remains further certification. The low bioavailability restricts most studies around PROTACs on cellular level and the evaluation of proteolysis is also inefficient^247^.

Despite facing significant challenges, E3 ligases VHL and CRBN are identified to be recruited by PROTACs. Wee is the serine/threonine kinase-family member and regulates the G2/M checkpoint of cancers. Selective degradation of Wee1 over other kinases is demonstrated and the PROTACs make adavosertib (WEE1 inhibitor) link to either the VHL ligand VH032 or to the CRBN ligand pomalidomide^248^. Therefore, it is of great significance to explore more E3 ligases and develop corresponding PROTACs.

In one word, UPS-targeted therapy is the promising method for pathological BME. However, current studies only have covered partial E3 ligases, which should be investigated more comprehensively and systematically to reveal mechanisms maintaining skeletal system homeostasis. Studies aiming to discover more unknown E3 ligases or mechanisms of unveiled E3 ligases should contribute to develop novel PROTAC drugs.

## Acknowledgments

This work was supported, in part, by the 10.13039/501100001809National Natural Science Foundation of China Grants (82022047 and 81972100), 10.13039/501100012166National Key Research and Development Program of China Grants (2019YFA0906001), Guangdong Provincial Science and Technology Innovation Council Grant (2017B030301018, China). We acknowledge the assistance of Core Research Facilities of Southern University of Science and Technology.

## Author contributions

Design of the review: Yuechao Dong and Huiling Cao; Draft the manuscript: Yuechao Dong and Yangshan Chen; Draw the figures: Yangshan Chen and Guixing Ma; Review and edit the manuscript: Huiling Cao and Yuechao Dong. All authors approved the final version of the manuscript.

## Conflicts of interest

The authors declare no conflicts of interest.
